# SARS-CoV-2 ORF6 Disrupts Bidirectional Nucleocytoplasmic Transport through Interactions with Rae1 and Nup98

**DOI:** 10.1128/mBio.00065-21

**Published:** 2021-04-13

**Authors:** Amin Addetia, Nicole A. P. Lieberman, Quynh Phung, Tien-Ying Hsiang, Hong Xie, Pavitra Roychoudhury, Lasata Shrestha, Michelle A. Loprieno, Meei-Li Huang, Michael Gale, Keith R. Jerome, Alexander L. Greninger

**Affiliations:** a Department of Laboratory Medicine and Pathology, University of Washington, Seattle, Washington, USA; b Molecular and Cellular Biology Graduate Program, University of Washington, Seattle, Washington, USA; c Center for Innate Immunity and Immune Disease, Department of Immunology, University of Washington School of Medicine, Seattle, Washington, USA; d Vaccine and Infectious Disease Division, Fred Hutchinson Cancer Research Center, Seattle, Washington, USA; Kyoto University; Columbia University/HHMI

**Keywords:** Nup98, ORF6, RNA virus, Rae1, SARS-CoV-2, VSV M, nucleocytoplasmic transport, virology

## Abstract

RNA viruses that replicate in the cytoplasm often disrupt nucleocytoplasmic transport to preferentially translate their own transcripts and prevent host antiviral responses. The *Sarbecovirus* accessory protein ORF6 has previously been shown to be a major inhibitor of interferon production in both severe acute respiratory syndrome coronavirus (SARS-CoV) and severe acute respiratory syndrome coronavirus 2 (SARS-CoV-2). Here, we show SARS-CoV-2-infected cells display an elevated level of nuclear mRNA accumulation compared to mock-infected cells. We demonstrate that ORF6 is responsible for this nuclear imprisonment of host mRNA, and using a cotransfected reporter assay, we show this nuclear retention of mRNA blocks expression of newly transcribed mRNAs. ORF6’s nuclear entrapment of host mRNA is associated with its ability to copurify with the mRNA export factors, Rae1 and Nup98. These protein-protein interactions map to the C terminus of ORF6 and can be abolished by a single amino acid mutation in Met58. Overexpression of Rae1 restores reporter expression in the presence of SARS-CoV-2 ORF6. SARS-CoV ORF6 also interacts with Rae1 and Nup98. However, SARS-CoV-2 ORF6 more strongly copurifies with Rae1 and Nup98 and results in significantly reduced expression of reporter proteins compared to SARS-CoV ORF6, a potential mechanism for the delayed symptom onset and presymptomatic transmission uniquely associated with the SARS-CoV-2 pandemic. We also show that both SARS-CoV and SARS-CoV-2 ORF6 block nuclear import of a broad range of host proteins. Together, these data support a model in which ORF6 clogs the nuclear pore through its interactions with Rae1 and Nup98 to prevent both nuclear import and export, rendering host cells incapable of responding to SARS-CoV-2 infection.

## Abstract (precis)

SARS-CoV-2, the causative agent of coronavirus disease 2019 (COVID-19), is an RNA virus with a large genome that encodes multiple accessory proteins. While these accessory proteins are not required for growth *in vitro*, they can contribute to the pathogenicity of the virus.

## INTRODUCTION

Severe acute respiratory syndrome coronavirus 2 (SARS-CoV-2), the causative agent of coronavirus disease 2019 (COVID-19), is a single-stranded RNA virus belonging to the *Betacoronavirus* genus (1). With their large genomes, coronaviruses, including SARS-CoV-2 encodes accessory proteins that are not required for viral replication but can contribute the virus’s pathogenicity (2).

The accessory protein ORF6 of SARS-CoV was previously shown to disable induction of interferon pathways by blocking nuclear import of STAT1 via interaction with nuclear import factors (3). SARS-CoV-2 ORF6 was recently shown to copurify with Rae1 and Nup98, providing a potential mechanism for interfering with nuclear transport (4). Nup98 is a component of the nuclear pore complex and interacts with the RNA export factor Rae1 to bind single-stranded RNA and facilitate the translocation of mRNA through the nuclear pore complex (5, 6).

Intriguingly, Nup98 and Rae1 are also targeted by the matrix (M) protein of vesicular stomatitis virus (VSV) and ORF10 of Kaposi’s sarcoma-associated herpesvirus (KSHV) to trap mRNA transcripts in the nucleus (7–9). A single methionine residue surrounded by acidic residues within both VSV M and KSHV ORF10 is critical for interactions with Rae1, and mutations at this residue impair the viral proteins’ ability to block mRNA nuclear export (4, 8, 10).

SARS-CoV-2 ORF6 contains a similar methionine in its C terminus that is surrounded by acidic residues, which may facilitate an interaction with the nucleic binding site of the Rae1·Nup98 complex (4). These interactions could plausibly disrupt nuclear mRNA export, in addition to the block in STAT1 nuclear import. Earlier work also demonstrated that SARS-CoV ORF6 downregulated expression of cotransfected constructs, leading to the potential of a bidirectional block of nucleocytoplasmic transport (11). Here, we investigated the impact of SARS-CoV and SARS-CoV-2 ORF6 on bidirectional nucleocytoplasmic transport in a variety of host cells.

## RESULTS

### SARS-CoV-2-infected cells accumulate mRNA in the nucleus.

**FIG 1:**
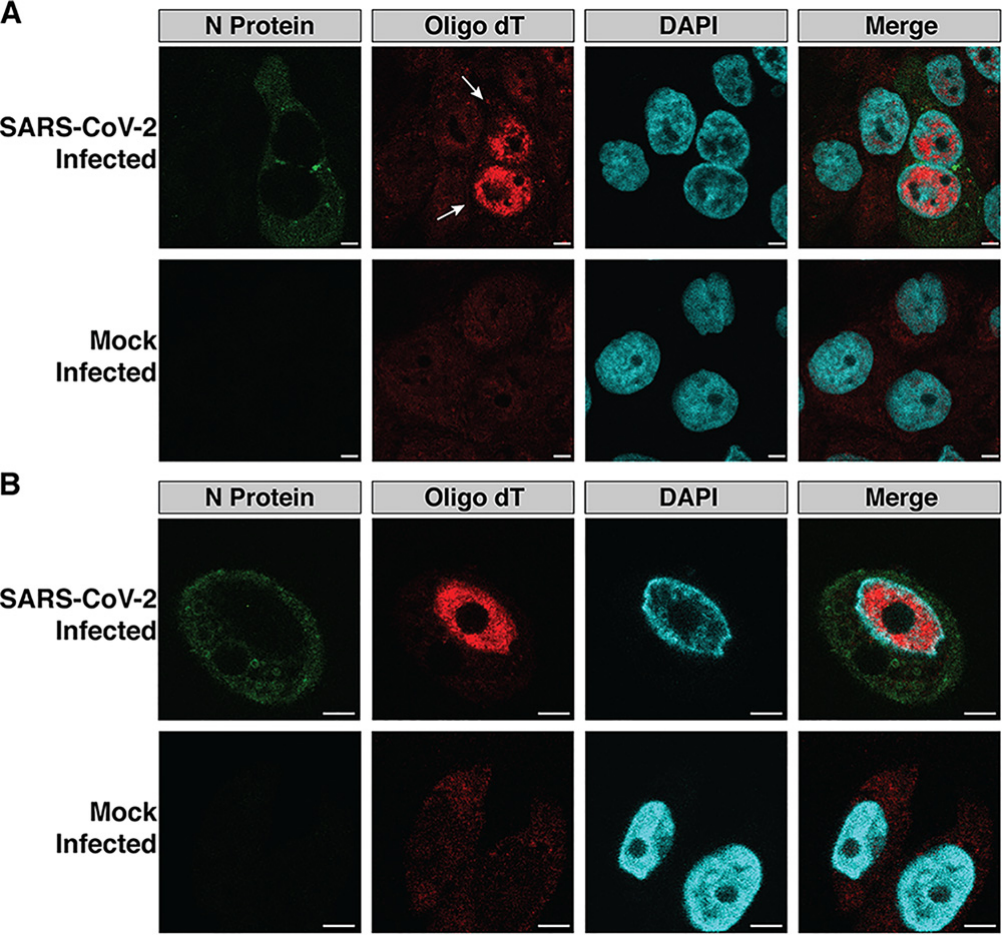
SARS-CoV-2-infected cells accumulate mRNA in the nuclei. (A and B) Calu3 (A) and HBEC3-ACE2 (B) cells were infected with SARS-CoV-2. Twenty-four hours postinfection, the cells were stained with an oligo(dT)(30) probe conjugated to an Alexa Fluor 594 fluorophore. Poly(A) mRNA was localized to the nuclei in infected cells (white arrows). mRNA was more evenly distributed throughout the nuclei and cytoplasm in mock and uninfected cells. Bars, 5 μm.

Numerous RNA viruses, including VSV and Zika virus, block host mRNA export in infected cells (7, 12). We examined whether SARS-CoV-2 similarly blocks nuclear export of host mRNA by infecting the human lung adenocarcinoma cell line, Calu3, and a human bronchial epithelial cell line stably expressing the SARS-CoV-2 receptor angiotensin-converting enzyme 2 (ACE2), HBEC3-ACE2, with SARS-CoV-2. Twenty-four hours postinfection, we examined the mRNA distribution in the SARS-CoV-2-infected and mock-infected cells (Fig. 1A and B; see also Fig. S1 in the supplemental material). In SARS-CoV-2-infected cells, mRNA was primarily localized to the nuclei, while the mock-infected cells displayed a more even distribution of mRNA in the nuclei and cytoplasm. This nuclear mRNA accumulation phenotype was observed in both SARS-CoV-2-infected Calu3 (Fig. 1A and Fig. S1) and HBEC3-ACE2 (Fig. 1B) cells.

10.1128/mBio.00065-21.1 FIG S1 Poly(A) mRNA staining of Calu3 cells infected with SARS-CoV-2 revealed nuclear localization of mRNA in infected cells (highlighted with white arrows). Scale bars, 20 μm. Download FIG S1, PDF file, 0.8 MB. Copyright © 2021 Addetia et al. 2021 Addetia et al. https://creativecommons.org/licenses/by/4.0/ This content is distributed under the terms of the Creative Commons Attribution 4.0 International license.

### SARS-CoV-2 ORF6 blocks nuclear export of host mRNA.

**FIG 2:**
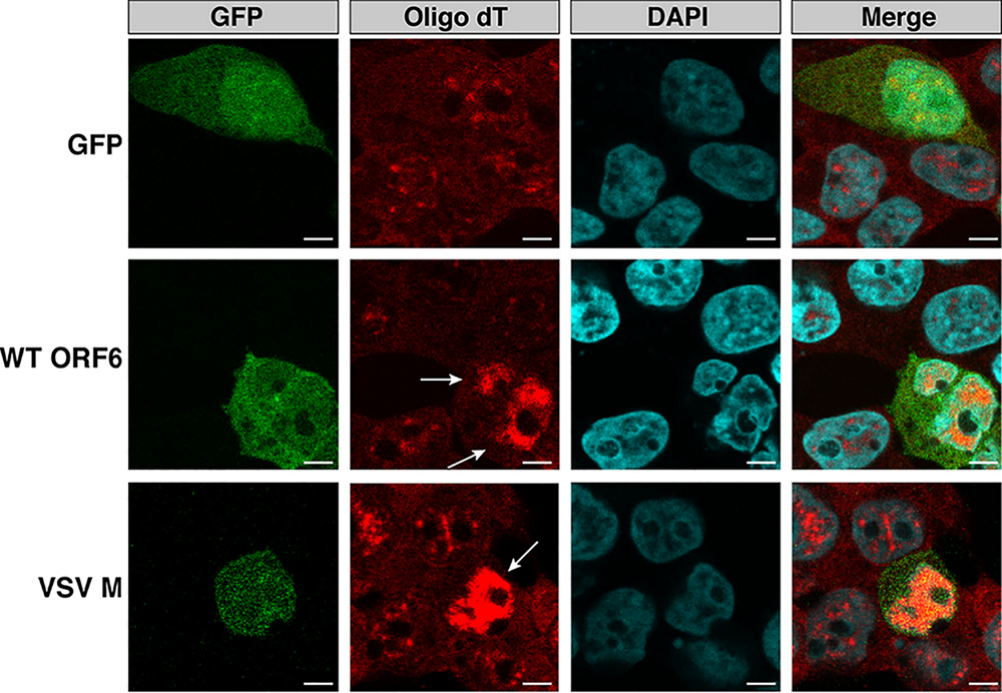
The SARS-CoV-2 accessory protein ORF6 is responsible for the nuclear mRNA accumulation phenotype observed in SARS-CoV-2-infected cells. 293T cells were transiently transfected with GFP, GFP-SARS-CoV-2 ORF6, or GFP-VSV M. Staining for poly(A) mRNA was conducted 24 h posttransfection. Cells expressing SARS-CoV-2 ORF6 or VSV M (white arrows) displayed an accumulation of mRNA in the nuclei, while those transfected with GFP displayed mRNA localization patterns identical to those of untransfected cells. Bars, 5 μm.

ORF6 interacts with the mRNA export factor Rae1 and the nuclear pore complex component Nup98 (4). VSV M and KSHV ORF10, which both interact with Rae1 and Nup98, produce an accumulation of mRNA in the nuclei of transfected cells (7, 9). We investigated whether ORF6 was responsible for the nuclear localization of mRNA observed during SARS-CoV-2 infection (Fig. 1A and B) by transiently transfecting human embryonic kidney, 293T, cells with either green fluorescent protein (GFP), GFP-tagged ORF6, or GFP-tagged VSV M. In cells transfected with GFP, mRNA was distributed throughout the cell, indistinguishable from the mRNA localization pattern in untransfected cells (Fig. 2). In contrast, mRNA in cells expressing wild-type (WT) ORF6 and VSV M was present in multiple foci within the nucleus, suggesting that the mRNA in these cells was accumulating in the nucleus (Fig. 2). Identical mRNA nuclear accumulation phenotypes were observed in Calu3 cells and the lung epithelial carcinoma cell line, A549, transiently transfected with ORF6 (Fig. S2A and B).

10.1128/mBio.00065-21.2 FIG S2 (A) A549 and (B) Calu3 cells were transiently transfected with GFP, GFP-SARSCoV-2 ORF6, or GFP-VSV M. Staining for poly(A) mRNA revealed cells expressing SARSCoV-2 ORF6 and VSV M accumulated mRNA in the nuclei. White arrows depict cells transfected with SARS-CoV-2 ORF6 or VSV M. Scale bars, 5 μm. Download FIG S2, PDF file, 2.7 MB. Copyright © 2021 Addetia et al. 2021 Addetia et al. https://creativecommons.org/licenses/by/4.0/ This content is distributed under the terms of the Creative Commons Attribution 4.0 International license.

### SARS-CoV-2 ORF6 downregulates protein expression of newly transcribed genes.

We next examined how the nuclear accumulation of host mRNA in cells expressing ORF6 affected host protein expression. We transiently transfected 293T cells with mCherry, mCherry-tagged ORF6, or mCherry-tagged VSV M and measured nascent protein expression in these cells 24 h posttransfection using a Click-iT labeling assay in which newly synthesized proteins incorporate l-azidohomoalanine instead of methionine. Nascent protein synthesis can then be quantified by labeling the l-azidohomoalanine residues with a fluorescent marker and compared across conditions by normalizing to the total number of cells labeled. Similar levels of nascent protein expression were observed in cells expressing mCherry (mean fluorescein isothiocyanate [FITC]/Hoechst ratio, 1.18), ORF6 (mean FITC/Hoechst ratio, 1.32; *P* = 0.31), and VSV M (mean FITC/Hoechst ratio, 1.25; *P* = 0.59) (Fig. S3), suggesting that ORF6 does not impact translation of existing cytoplasmic mRNA transcripts and likely blocks expression of only newly transcribed mRNA transcripts.

10.1128/mBio.00065-21.3 FIG S3 Nascent protein synthesis was measured in cells transfected with mCherry, SARS-CoV-2 ORF6, or VSV M and puromycin-treated cells using the Click-iT AHA Alexa Fluor 488 Protein Synthesis HCS Assay. Similar levels of nascent protein synthesis were observed in mCherry-, SARS-CoV-2 ORF6-, and VSV M-expressing cells. Download FIG S3, PDF file, 0.04 MB. Copyright © 2021 Addetia et al. 2021 Addetia et al. https://creativecommons.org/licenses/by/4.0/ This content is distributed under the terms of the Creative Commons Attribution 4.0 International license.

**FIG 3:**
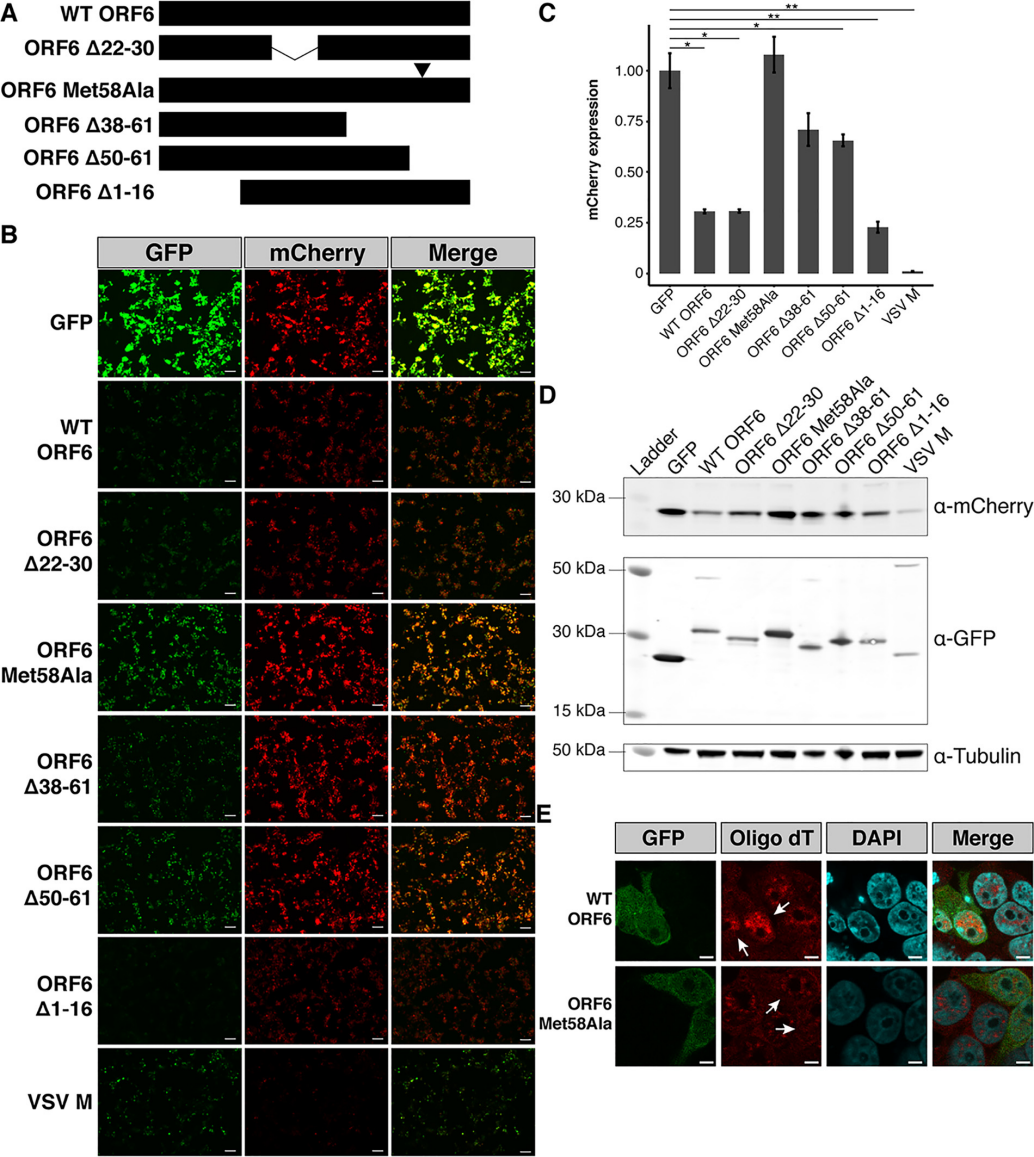
ORF6 of SARS-CoV-2 results in reduced mCherry reporter protein expression in 293T cells. (A) Schematic representation of ORF6 constructs used in this study. (B) 293T cells were transiently transfected with GFP-tagged constructs and mCherry and visualized 48 h after transfection. All images were taken with identical fluorescence gain settings. Bars, 100 μm. (C) The fluorescent intensities for three fields per cotransfection were measured with ImageJ and displayed as means ± standard errors (error bars). Wild-type (WT) ORF6 caused a significant reduction in mCherry expression. ORF6 constructs with deletions or a single amino acid substitution (Met58Ala) in the C terminus showed a smaller reduction in mCherry expression than WT ORF6. Cell lysates were collected for each of the conditions. (D) Western blotting confirmed the role of the C terminus of ORF6 in reducing protein expression in transfected cells. (E) Poly(A) mRNA staining of transiently transfected 293T cells revealed ORF6 Met58Ala-expressing cells do not accumulate mRNA in the nuclei, consistent with the results from the mCherry cotransfection assay. Bars, 5 μm. *, *P* < 0.05; **, *P* < 0.01.

For VSV M and KSHV ORF10, which both prevent nuclear export of mRNA, downregulation of expression from newly transcribed transcripts has been measured using luminescent and fluorescent reporter assays (7, 9). In these assays, cells are concurrently transfected with the viral protein and reporter constructs. Cells expressing the viral protein display a marked reduction in reporter expression, as the newly transcribed reporter transcripts are largely retained in the nuclei, inaccessible to the cell’s translational machinery. To assess whether ORF6’s blockage of nuclear export of mRNA similarly results in a reduction of newly transcribed transcripts and to map the residues critical for the nuclear accumulation of mRNA, we constructed a series of N-terminal GFP-tagged ORF6 constructs (Fig. 3A). We included a mutant ORF6 protein, ORF6 Δ22-30, which has independently arisen in multiple clinical SARS-CoV-2 strains and in a serially passaged culture SARS-CoV-2 isolate (see Table S1 and Fig. S4A to C in the supplemental material). We then cotransfected 293T cells with these ORF6 constructs and a reporter plasmid encoding mCherry.

10.1128/mBio.00065-21.4 FIG S4 Multiple clinical SARS-CoV-2 isolates and a serially passaged cultured SARSCoV-2 isolate contain a 9-amino-acid deletion in ORF6. (A) Schematic representation of the 9-amino-acid deletion in ORF6 identified through whole-genome sequencing of the SARS-CoV-2 clinical isolate, WA-UW-4572. (B) Reverse transcription-PCR (RT-PCR) with primers spanning ORF6 yielded a 452-bp PCR product for WA-UW-4572 rather than a 479-bp product confirming the ORF6 deletion in WA-UW-4572. (C) Six other clinical isolates and a cultured isolate with an identical deletion in ORF6 were identified by analyzing the ORF6 sequences of 67,000 publicly available SARSCoV-2 genomes. The isolates were genetically distinct and belonged to both major SARSCoV-2 lineages. Download FIG S4, PDF file, 0.3 MB. Copyright © 2021 Addetia et al. 2021 Addetia et al. https://creativecommons.org/licenses/by/4.0/ This content is distributed under the terms of the Creative Commons Attribution 4.0 International license.

10.1128/mBio.00065-21.7 TABLE S1 Clinical and cultured SARS-CoV-2 isolates with a 9-amino-acid deletion in ORF6 identified by analyzing ORF6 sequences from over 67,000 SARS-CoV-2 strains (https://www.gisaid.org/; accessed 17 July 2020). Download Table S1, DOCX file, 0.01 MB. Copyright © 2021 Addetia et al. 2021 Addetia et al. https://creativecommons.org/licenses/by/4.0/ This content is distributed under the terms of the Creative Commons Attribution 4.0 International license.

Similar to VSV M, cells expressing the GFP-ORF6 construct showed a significant reduction in mCherry expression (mean fluorescence intensity [MFI], 0.31; standard error [SE], 0.01; *P* = 0.01) relative to the cells transfected with GFP (MFI, 1.0; SE, 0.09) (Fig. 3B and C). Subsequent Western blotting (Fig. 3D) further confirmed mCherry expression was downregulated in cells expressing WT ORF6. A similar reduction of mCherry expression was observed in A549 cells transiently transfected with WT ORF6 (Fig. S5A and B).

10.1128/mBio.00065-21.5 FIG S5 (A) A549 cells were transiently transfected with GFP-tagged constructs and mCherry. Cells were visualized 24 h after transfection, and all images were taken with identical fluorescence gain settings. Scale bars, 100 μm. (B) Fluorescent intensities across three fields per conditions were measured with ImageJ and displayed as means ± standard errors. Staining for poly(A) mRNA in A549 (C) and Calu3 (D) cells revealed SARS-CoV-2 ORF6 Met58Ala-expressing cells do not accumulate mRNA in the nuclei, consistent with the results of the mCherry cotransfection assay. White arrows indicate transfected cells. Scale bars, 5 μm. *, *P* < 0.05; **, *P* < 0.01. Download FIG S5, PDF file, 2.0 MB. Copyright © 2021 Addetia et al. 2021 Addetia et al. https://creativecommons.org/licenses/by/4.0/ This content is distributed under the terms of the Creative Commons Attribution 4.0 International license.

ORF6 constructs containing deletions in the protein’s N terminus, ORF6 Δ1-16 (MFI, 0.23; SE, 0.03) and the clinical isolate variant ORF6 Δ22-30 (MFI, 0.31; SE, 0.01), displayed a three- to fourfold reduction in mCherry expression (Fig. 3B and C) similar to WT ORF6, indicating that the N terminus of ORF6 is not involved in downregulating protein expression. In contrast, mCherry expression was reduced 1.4- to 1.5-fold only in the presence of ORF6 constructs with deletions in the C terminus, ORF6 Δ38-61 (MFI, 0.71; SE, 0.08) and Δ50-61 (MFI, 0.66; SE, 0.03) (Fig. 3B and C).

In VSV M, a motif consisting of a methionine residue surrounded by acidic residues is critical for reducing expression levels of cotransfected reporters. The methionine residue within the motif is conserved between VSV M and KSHV ORF10, and a similar motif with a methionine residue is present in the SARS-CoV-2 ORF6 C terminus (Fig. S4A). We changed this methionine residue in ORF6 to an alanine, generating the construct ORF6 Met58Ala (Fig. 2A). Transfection of ORF6 Met58Ala did not downregulate mCherry expression (MFI, 1.08; SE, 0.09) (Fig. 3B and C), suggesting that Met58 is critical for the function of ORF6. We then validated that the observed increase in mCherry expression in cells transfected with ORF6 Met58Ala compared to cells transfected with WT ORF6 was attributed to differences in mRNA localization. Staining of mRNA in transiently transfected 293T (Fig. 3E), A549 (Fig. S5C), and Calu3 (Fig. S5D) cells revealed distinct mRNA localization patterns in WT ORF6- and ORF6 Met58Ala-transfected cells. Unlike WT ORF6-expressing cells, ORF6 Met58Ala-expressing cells did not display an accumulation of mRNA in the nucleus, confirming the importance of Met58 to the functioning of ORF6.

### The C terminus of SARS-CoV-2 ORF6 interacts with Rae1 and Nup98.

**FIG 4:**
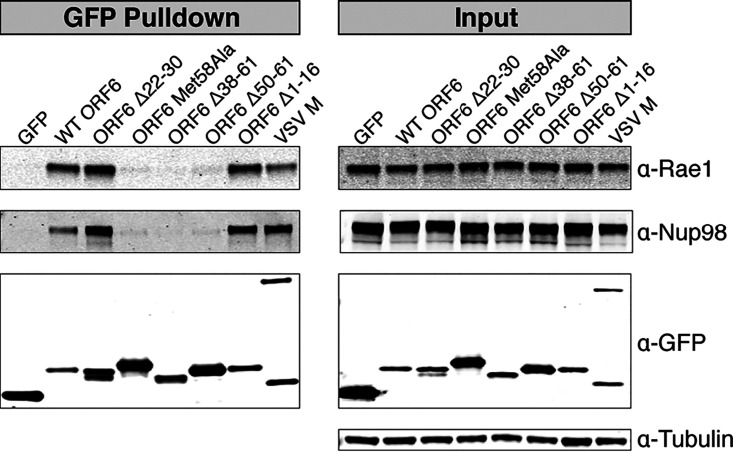
Affinity purification of GFP-tagged constructs. 293T cells were transiently transfected with GFP-tagged constructs. Forty-eight hours after transfection, the GFP-tagged proteins were rapidly captured using an anti-GFP resin. Western blotting revealed that ORF6 interacts with the mRNA nuclear export factor Rae1 and the nuclear pore complex protein Nup98. ORF6 constructs with C-terminal deletions or a substitution did not pull down Rae1 or Nup98.

In VSV M and KSHV ORF10, downregulation of cotransfected fluorescent and luminescent reporters and impairment of mRNA nuclear export occur due to interactions with the nuclear mRNA export factor Rae1 and nuclear pore complex component Nup98 (7, 9). VSV M displaces single-stranded RNA in the Rae1·Nup98 complex to prevent nuclear export of host mRNA (8). We hypothesized the inability of the ORF6 C-terminal deletions to downregulate mCherry expression in a manner similar to that of WT ORF6 (Fig. 3B to D) was attributed to the loss of the interaction between these ORF6 constructs and Rae1 and Nup98. We transfected 293T cells with GFP-tagged ORF6 constructs (Fig. 3A) and rapidly affinity purified the GFP-tagged proteins. Western blotting on the eluates confirmed that WT ORF6, along with ORF6 constructs with N-terminal deletions, interacts with Rae1 and Nup98 (Fig. 4). The C-terminal deletion constructs, ORF6 Δ38-61 and ORF6 Δ50-61, did not pull down Rae1 or Nup98 (Fig. 4). These data suggest that the C terminus of ORF6 interacts with Rae1 and Nup98, while the N terminus is not essential for the observed interactions. This is consistent with the observation that C-terminal deletion mutants of ORF6 did not dramatically reduce expression of the mCherry reporter (Fig. 3B to D).

The methionine residue in the Rae1-Nup98 interacting motif of VSV M forms multiple intermolecular interactions with amino acid residues in the nucleic acid binding site of Rae1 and facilitates the interaction between VSV M and the Rae1·Nup98 complex (8). We hypothesized that Met58 of SARS-CoV-2 ORF6 is similarly responsible for interactions with Rae1 and Nup98. Affinity purification of ORF6 Met58Ala revealed that it does not interact with Rae1 or Nup98 (Fig. 4), confirming the importance of Met58 in the ORF6-Rae1 and ORF6-Nup98 interactions.

### Overexpression of Rae1 restores mCherry reporter expression in cells transfected with ORF6.

**FIG 5:**
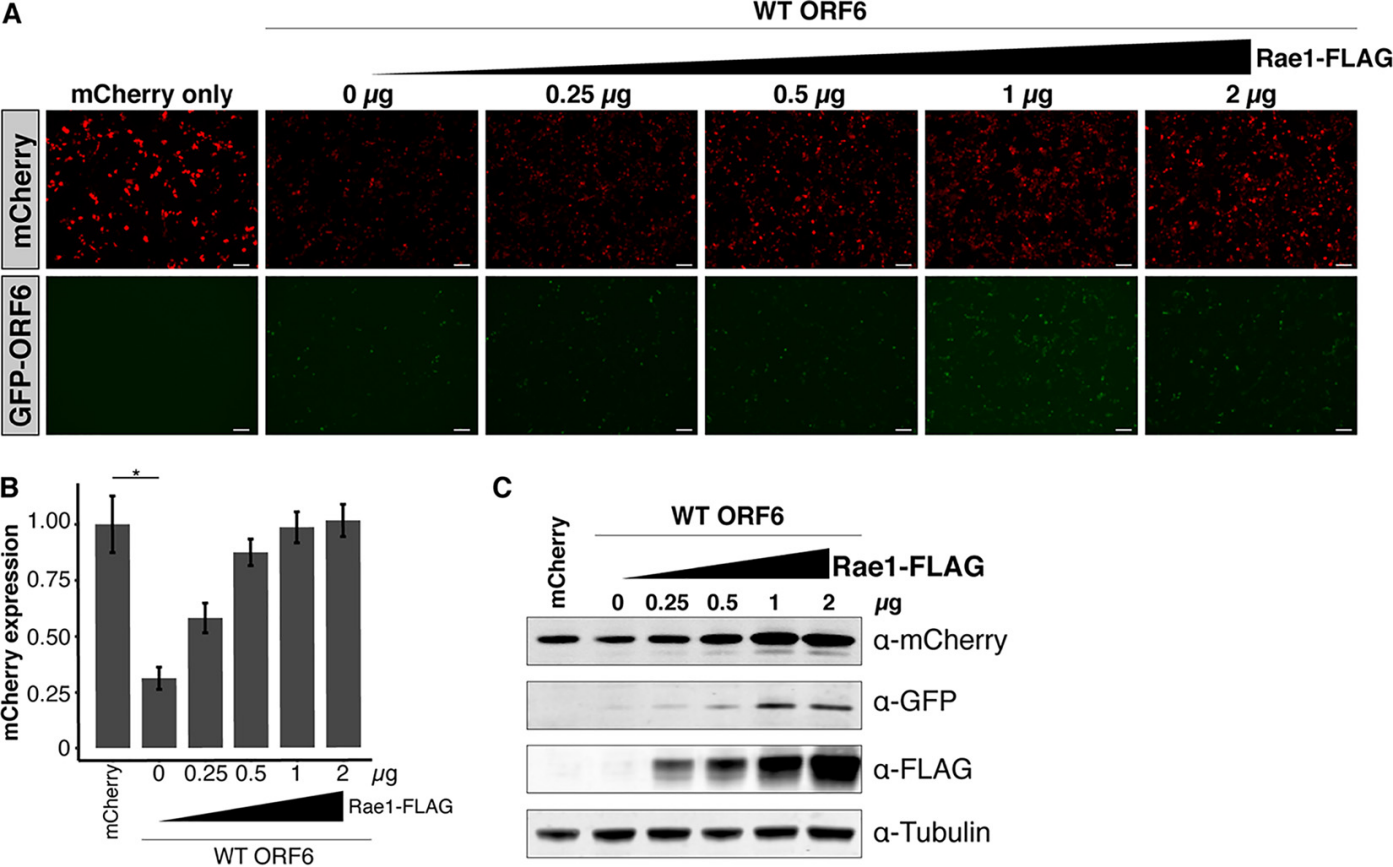
Overexpression of Rae1 rescues mCherry expression in cells transfected with ORF6. (A) 293T cells were cotransfected with equal amounts GFP-ORF6 and mCherry and an increasing amount of Rae1. Bars, 100 μm. (B) Expression of the fluorescent reporters was visualized and quantified 48 h after transfection. In the presence of ORF6, mCherry expression was restored in a dose-dependent manner. (C) Western blotting confirmed that mCherry expression was rescued in a dose-dependent manner with increasing Rae1-FLAG. *, *P* < 0.05.

We next investigated whether we could restore mCherry expression in 293T cells transfected with ORF6 by overexpressing Rae1. Rae1 overexpression restored mCherry expression in a dose-dependent manner (Fig. 5A and B). Subsequent Western blotting confirmed this Rae1 dose-dependent rescue of mCherry expression (Fig. 5C). These data indicate that ORF6’s interaction with Rae1 is responsible for downregulating mCherry reporter expression in cell culture.

### SARS-CoV-2 ORF6 more strongly copurifies with Rae1 and Nup98 compared to SARS-CoV ORF6.

**FIG 6:**
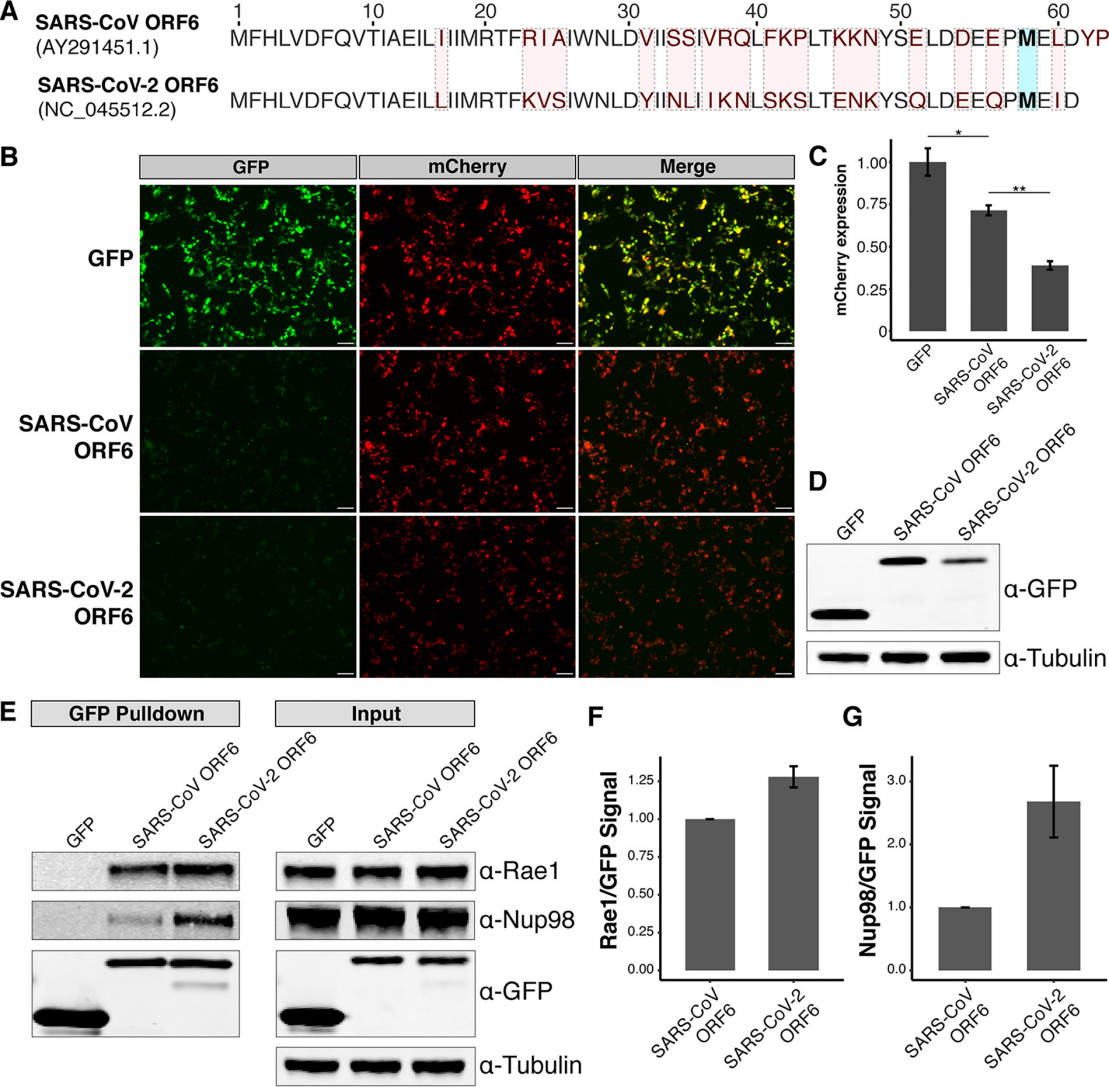
SARS-CoV-2 ORF6 represses reporter expression and copurifies with relatively more Rae1-Nup98 than SARS-CoV ORF6. (A) Comparison between the amino acid sequences of ORF6 of SARS-CoV and ORF6 of SARS-CoV-2. Residues differing between the two viruses are highlighted in red. The residue (Met58) implicated in binding the Rae1·Nup98 complex is highlighted in blue. (B) 293T cells were transiently transfected with GFP-tagged constructs and mCherry and visualized 24 h after transfection. Bars, 100 μm. (C) Cells transfected with SARS-CoV-2 ORF6 showed significantly reduced mCherry expression compared to those transfected with SARS-CoV ORF6. (D) Western blotting showed decreased expression of SARS-CoV-2 ORF6 compared to SARS-CoV ORF6 in 293T cells. (E) Affinity purification of GFP-tagged constructs demonstrates both ORF6 of SARS-CoV and ORF6 of SARS-CoV-2 interact with Rae1 and Nup98. (F and G) Densitometry shows SARS-CoV-2 ORF6 copurifies with relatively more Rae1 (F) and Nup98 (G) compared to SARS-CoV ORF6. *, *P* < 0.05; **, *P* < 0.01.

We next compared the relative ability of SARS-CoV ORF6 and SARS-CoV-2 ORF6 to downregulate reporter expression. SARS-CoV ORF6 and SARS-CoV-2 ORF6 share 69% identity by amino acid, including the same methionine residue surrounded by acidic residues (Fig. 6A). SARS-CoV ORF6 has been shown to downregulate expression of a cotransfected construct in a dose-dependent manner (11), suggesting that its C terminus may also interact with the Rae1·Nup98 complex.

We cotransfected 293T cells with GFP-tagged SARS-CoV ORF6 or GFP-tagged SARS-CoV-2 ORF6 and mCherry to assess the impact of these constructs on protein expression. Compared to cells transfected with GFP alone, cells transfected with SARS-CoV ORF6 displayed reduced mCherry expression (MFI of 1 and SE of 0.08 versus MFI of 0.71 and SE of 0.03); however, this difference was not significant (*P* = 0.06) (Fig. 6B and C). Cells transfected with SARS-CoV-2 ORF6 displayed a significant reduction in mCherry expression compared to cells transfected with SARS-CoV ORF6 (MFI, 0.3; SE, 0.02; *P* = 0.001) (Fig. 6B and C). Western blotting also demonstrated decreased expression of SARS-CoV-2 ORF6 relative to SARS-CoV ORF6, suggesting that expression levels do not explain the differential effects on reporter gene expression (Fig. 6D).

We hypothesized the differences in mCherry expression between SARS-CoV ORF6 and SARS-CoV-2 ORF6 could be attributed to differences in copurification of Rae1 and Nup98. We transfected 293T cells with the GFP-tagged constructs and affinity purified the tagged proteins. Western blotting revealed that SARS-CoV ORF6 interacts with Rae1 and Nup98, similar to SARS-CoV-2 ORF6 (Fig. 6E). Densitometry on the ratio of prey to bait demonstrated that SARS-CoV-2 ORF6 copurified with 1.3-fold more Rae1 (Fig. 6F) and 2.7-fold more Nup98 (Fig. 6G) compared to SARS-CoV ORF6. These data suggest that SARS-CoV-2 ORF6 may more dramatically repress protein expression via a stronger interaction with the Rae1·Nup98 complex compared to SARS-CoV ORF6.

Next, we examined the sequence variation of ORF6 across the *Sarbecovirus* subgenus. Three distinct clades of sarbecoviruses have been described thus far with SARS-CoV and SARS-CoV-2 belonging to clade 1 and clade 2, respectively. The ORF6 protein in the clade 1 sarbecoviruses is two amino acid residues longer than the ORF6 protein in the clade 2 sarbecoviruses (Fig. 6A and Fig. S6A) (13). Notably, the protein sequence of ORF6 in the clade 2 Rhinolophus affinis sarbecovirus RaTG13 (GenBank accession no. MN996532.2) is more similar to that in the clade 2 pangolin sarbecovirus Pangolin-CoV/Guangdong/1/2019 (EPI_ISL_410721) compared to that in the clade 1 Rhinolophus affinis sarbecovirus LYRa11 (GenBank accession no. KF569996.1). This suggests that sequence variation in ORF6 is unrelated to the zoonotic host of sarbecoviruses, consistent with Rae1 and Nup98 being highly conserved across eukaryotes (6).

10.1128/mBio.00065-21.6 FIG S6 (A) An analysis of the ORF6 homologs in the related zoonotic sarbecoviruses, LYRa11 (GenBank accession no. KF569996.1), Pangolin-CoV (EPI_ISL_410721), and RaTG13 (MN996532.2), reveals that the ORF6 protein in the clade 1 sarbecoviruses is 2 amino acids longer than that in the clade 2 sarbecoviruses. The clade 3 sarbecovirus BM48-31 (GenBank accession no. NC_014470.1) was used as an outgroup in the phylogenetic analysis. Residues different from SARS-CoV-2 ORF6 are highlighted in red, while the critical methionine residue is highlighted in blue. (B) 293T cells were transiently transfected with GFP-tagged constructs and mCherry. Expression was visualized 48 h after transfection, and fluorescent intensities across three fields per experimental condition were measured for SARS-CoV ORF6 constructs (C) and SARS-CoV-2 ORF6 constructs (D). Scale bars, 100 μm. *, *P* < 0.05; **, *P* < 0.01. (E) Expression levels of the GFP-tagged SARS-CoV and SAR-CoV-2 ORF6 constructs were visualized by Western blotting. Download FIG S6, PDF file, 1.7 MB. Copyright © 2021 Addetia et al. 2021 Addetia et al. https://creativecommons.org/licenses/by/4.0/ This content is distributed under the terms of the Creative Commons Attribution 4.0 International license.

To determine whether the difference in sequence length between SARS-CoV ORF6 and SARS-CoV-2 ORF6 impacted the differential ability of these proteins to downregulate mCherry expression, we generated a SARS-CoV ORF6 mutant construct, SARS-CoV ORF6 Δ62-63, in which the last 2 amino acids were deleted and a SARS-CoV-2 ORF6 mutant construct, SARS-CoV-2 ORF6 +62-63, in which the last 2 amino acids of SARS-CoV ORF6 were added to the C terminus of SARS-CoV-2 ORF6. Next, we repeated the mCherry cotransfection reporter assay with these constructs (Fig. S6B). The mCherry expression was significantly higher in cells transfected with SARS-CoV ORF6 Δ62-63 (MFI, 1.28; SE, 0.08; *P* = 0.048) compared to SARS-CoV WT ORF6 (MFI, 1; SE, 0.04) (Fig. S6C). Cells transfected with SARS-CoV-2 ORF6 +62-63 (MFI, 1.16; SE, 0.04) displayed higher mCherry expression than those transfected with SARS-CoV-2 WT ORF6 (MFI, 1; SE, 0.13), however, the difference in mCherry expression between the conditions was not significant (*P* = 0.34) (Fig. S6D). We then investigated whether the Met58 is critical to the function of SARS-CoV ORF6. The mCherry expression in cells transfected with SARS-CoV ORF6 Met58Ala (MFI, 1.38; SE, 0.05; *P* = 0.005) was significantly higher than that in cells transfected with SARS-CoV WT ORF6, indicating that Met58 is critical for the functioning of both SARS-CoV ORF6 and SARS-CoV-2 ORF6 (Fig. S6B to E).

### SARS-CoV and SARS-CoV-2 ORF6 block nuclear import of a broad range of host factors.

**FIG 7:**
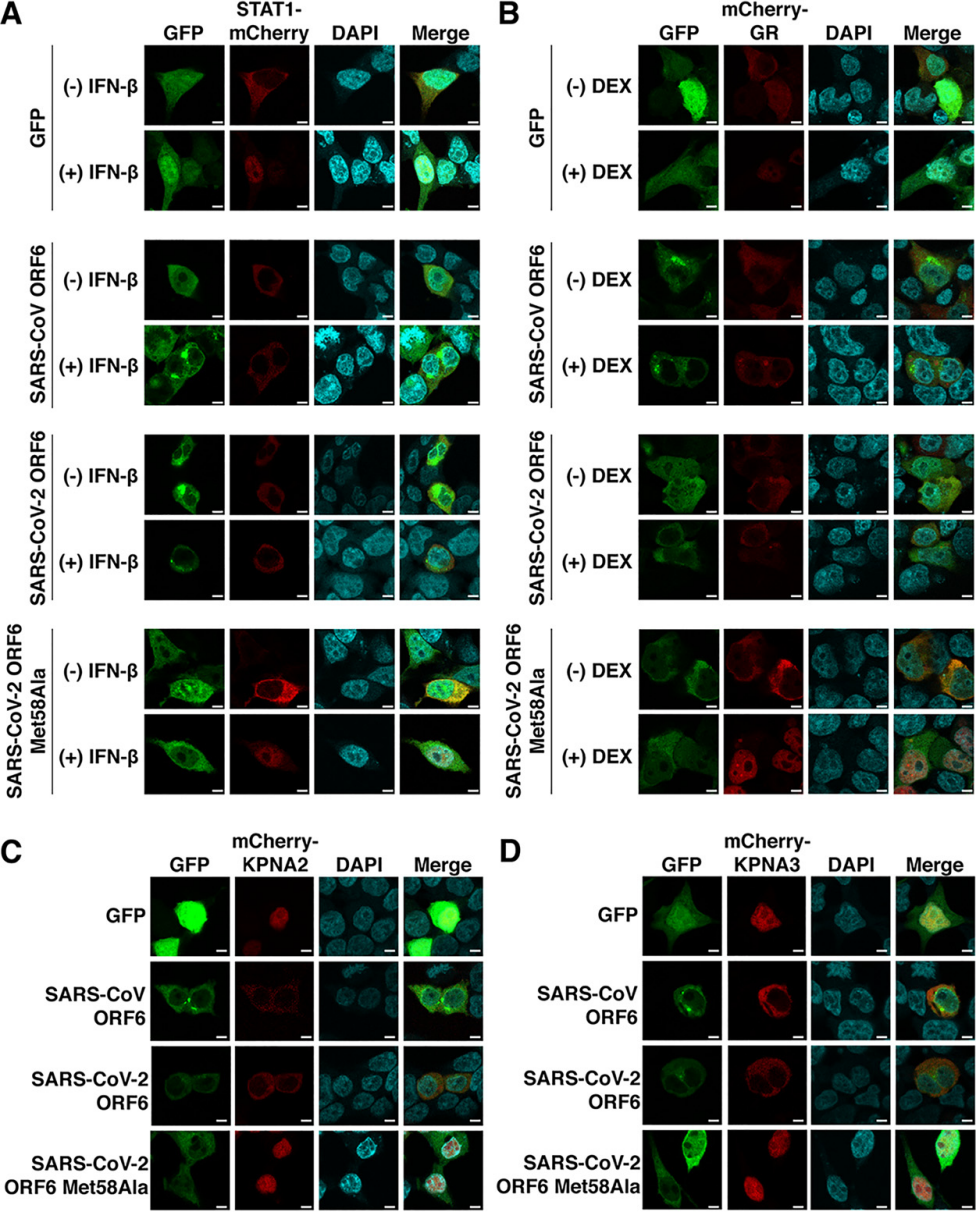
SARS-CoV ORF6 and SARS-CoV-2 ORF6 inhibit nuclear import of a broad range of host factors. (A) 293T cells were cotransfected with equal amounts of GFP-tagged constructs and STAT1 with an mCherry tag. STAT1 localized to the nuclei following IFN-β stimulation in GFP- and SARS-CoV-2 ORF6 Met58Ala-expressing cells but remained in the cytoplasm in SARS-CoV ORF6- and SARS-CoV-2 ORF6-expressing cells. (B) 293T cells were cotransfected with GFP-tagged constructs and mCherry-tagged glucocorticoid receptor (GR). The glucocorticoid receptor was translocated into the nuclei following dexamethasone (DEX) stimulation in GFP- and SARS-CoV-2 ORF6 Met58Ala-expressing cells yet remained in the cytoplasm in SARS-CoV ORF6- and SARS-CoV-2-expressing cells. (C and D) The importins KPNA2 (C) and KPNA3 (D) displayed distinct localization patterns based on the GFP-tagged constructs that was transfected with the mCherry-tagged importin. In GFP- and SARS-CoV-2 ORF6 Met58Ala-expressing cells, the importins localized to the nuclei, while in SARS-CoV ORF6- and SARS-CoV-2 ORF6-expressing cells, the importins were localized to the cytoplasm. Bars, 5 μm.

Both SARS-CoV and SARS-CoV-2 have been demonstrated to block nuclear import of the transcription factor, STAT1, during infection in cell culture (3, 14). Consistent with previous reports, we found that nuclear import of STAT1 was impaired in cells expressing either SARS-CoV ORF6 or SARS-CoV-2 ORF6 (Fig. 7A). STAT1 accumulated in the nuclei following interferon beta (IFN-β) stimulation in cells expressing GFP or SARS-CoV-2 ORF6 Met58Ala (Fig. 7A) but remained in the cytoplasm after IFN-β stimulation in cells expressing SARS-CoV ORF6 or SARS-CoV-2 ORF6.

We reasoned that the blockade of nuclear import was unlikely to be specific to STAT1. The transcription factor glucocorticoid receptor (GR) is shuttled into the nucleus following stimulation with a steroid through interactions with importin β and the nucleoporin Nup62 (15). In cells expressing GFP or SARS-CoV-2 ORF6 Met58Ala (Fig. 7B), GR was translocated into the nuclei following dexamethasone stimulation. However, GR remained in the cytoplasm after dexamethasone stimulation in cells expressing SARS-CoV ORF6 or SARS-CoV-2 ORF6 (Fig. 7B), consistent with a broad blockade of nuclear import by ORF6. We then investigated how SARS-CoV ORF6 and SARS-CoV-2 ORF6 impacted the localization patterns of the importins KPNA2 and KPNA3. These importins bind cargo proteins and facilitate translocation of their cargo into the nuclei. KPNA2 and KPNA3 were nuclear localized in cells expressing GFP or SARS-CoV-2 ORF6 Met58Ala (Fig. 7C and D). In contrast, both KPNA2 and KPNA3 localized to the cytoplasm in cells expressing SARS-CoV ORF6- and SARS-CoV-2 ORF6, suggesting that ORF6, through its interactions with the Rae1·Nup98 complex, clogs the nuclear pore, preventing nuclear import of a broad array of host factors.

## DISCUSSION

Here, we demonstrate that SARS-CoV-2 enacts a bidirectional block of nucleocytoplasmic transport at the nuclear pore, preventing both mRNA export from and stimulus-dependent host protein import into the nuclei of infected cells. We show the accessory protein ORF6 is responsible for this nuclear imprisonment of mRNA, which further results in downregulation of expression of new transcribed transcripts. Inhibition of mRNA nuclear export by ORF6 is attributed to its interactions with the mRNA nuclear export factor Rae1 and the nuclear pore complex component Nup98. We demonstrate that inhibition of mRNA nuclear export, reporter repression, and the host-virus protein-protein interactions is critically dependent on a methionine residue in the ORF6 C terminus. Additionally, we show an ORF6 allele with a 9-amino-acid deletion that has arisen in multiple clinical SARS-CoV-2 isolates and a serially passaged culture isolate maintains the ability to downregulate expression of cotransfected reporter and interact with Rae1 and Nup98. We find that SARS-CoV-2 ORF6 more strongly represses reporter expression and more strongly copurifies with Rae1-Nup98 compared to SARS-CoV ORF6. Finally, we show that both SARS-CoV and SARS-CoV-2 ORF6 inhibit nuclear import of a broad range of host factors, including those that interact with nucleoporins besides Nup98. Together, these data indicate that the *Sarbecovirus* accessory protein ORF6 prevents bidirectional nucleocytoplasmic transport through its interactions with Rae1 and Nup98, leaving host cells incapable of responding to viral infection.

RNA viruses, including coronaviruses, that replicate in the cytoplasm have mechanisms to suppress cellular translation, which allows these viruses to use the host’s translational machinery to preferentially express viral proteins (16–18). In SARS-CoV, ORF6 is not required for growth *in vitro*; however, expression of SARS-CoV ORF6 can increase the replication kinetics of SARS-CoV and the related murine hepatitis virus *in vitro* (19, 20). In addition, recombinant SARS-CoV isolates containing ORF6 grow to higher viral loads than recombinant isolates lacking ORF6 (19). This enhancement in viral growth could be attributed to both SARS-CoV ORF6’s ability to prevent host antiviral responses to viral infection via nuclear import or to blockade of nuclear export of newly transcribed mRNAs. As SARS-CoV-2 ORF6 similarly interacts with Rae1 and Nup98, we speculate that ORF6 is required for optimal growth of SARS-CoV-2.

In addition to enhancing viral replication, preventing bidirectional nucleocytoplasmic transport doubly suppresses the host antiviral response (16–18). The ability of the M protein of VSV to bind Rae1 and Nup98 and prevent mRNA nuclear export is associated with suppressed interferon-β gene expression (21). Furthermore, VSV strains containing a mutation at the residue responsible for the VSV M-Rae1-Nup98 interactions induce significantly higher interferon-α protein levels than strains containing wild-type alleles of the M protein (22). SARS-CoV-2 ORF6 has been shown to be an interferon antagonist (23) and likely downregulates both the induction of antiviral genes and the export of their mRNAs.

Beyond interfering with interferon expression by restricting nuclear export of mRNA, SARS-CoV-2 ORF6 acts as an interferon antagonist by preventing nuclear import of the transcription factor STAT1 (14, 24, 25). The results of previous studies have suggested that SARS-CoV ORF6 similarly blocks STAT1 nuclear import by sequestering KPNA2 in the cytoplasm (3); however, recent work has argued that SARS-CoV-2 ORF6 prevents STAT1 nuclear import by preventing docking at Nup98 (14). Our results further suggest that SARS-CoV-2’s blockage of nuclear import extends to additional host factors and extends to nuclear export. Our results support a model in which the interaction between SARS-CoV-2 ORF6 and the Rae1·Nup98 complex clogs the nuclear pore to prevent bidirectional nucleocytoplasmic transport of a broad array of factors. Together, our demonstration that SARS-CoV-2 ORF6 blocks both nuclear export of host mRNA and nuclear import of various host factors suggests that SARS-CoV-2-infected cells are likely incapable of responding to viral infection, consistent with SARS-CoV-2-infected cells displaying reduced expression of transcriptionally activated genes (26).

To date, SARS-CoV-2 has caused several thousand-fold more infections than SARS-CoV in part due to the distinct clinical presentations between the two viruses. COVID-19 patients display peak viral loads and maximum infectivity upon the onset of symptoms rather than after the onset of symptoms which is typical in patients with SARS (27). Furthermore, asymptomatic transmission was infrequently reported for SARS-CoV (28, 29); however, presymptomatic and asymptomatic transmission have been a defining challenge of the current SARS-CoV-2 pandemic (30–32). An important scientific challenge is defining the virological basis for these radically different infection profiles despite their close homology. Both the delayed onset of clinical symptoms and presymptomatic and asymptomatic transmission of SARS-CoV-2 could be attributed to increased potency of interferon antagonization in SARS-CoV-2 compared to SARS-CoV. ORF6 has already been shown to be a major interferon antagonist in both SARS-CoV and SARS-CoV-2 (3, 23, 33). ORF6 is one of the least similar accessory proteins (69% identical by amino acid) between the two viruses. Coupled with our demonstration of SARS-CoV-2 ORF6 more strongly downregulating protein expression and copurifying with more Rae1 and Nup98 than SARS-CoV ORF6, the differences between SARS-CoV ORF6 and SARS-CoV-2 ORF6 could explain at least some of the differences in clinical presentations between SARS and COVID-19.

Large-scale SARS-CoV-2 genomic surveillance projects have demonstrated that deletions can arise within the accessory genes of SARS-CoV-2 (34–36). Notably, none of these deletions have arisen in multiple SARS-CoV-2 lineages through multiple independent genomic rearrangement events. Our identification of seven unrelated clinical isolates with the same ORF6 deletion suggests that this deletion may be repeatedly selected for in SARS-CoV-2. This is further evidenced by the identification of a cultured SARS-CoV-2 that acquired the same deletion after successive passages in Vero cells (37). Similar to wild-type ORF6 allele, the clinical allele, ORF6 Δ22-30, can repress expression of a cotransfected reporter and still retains the Rae1·Nup98 interacting motif of ORF6. Further work is required to understand the functional role of the ORF6 N terminus and determine the selective pressures that are repeatedly selecting for the observed deletion.

Our study has a number of limitations. We relied on an mCherry reporter assay to measure ORF6’s impact on expression of newly transcribed transcripts. As such, our results may not perfectly reflect the degree to which expression of newly transcribed host transcripts is downregulated by ORF6 or during SARS-CoV-2 infection. More comparative work between SARS-CoV-2 and SARS-CoV ORF6 is needed in the context of viral replication. It would be intriguing to swap ORF6 between SARS-CoV and SARS-CoV-2 isolates to test the hypothesis that ORF6 is the major determinant of interferon antagonization and delayed symptom onset in animal models of SARS-CoV-2.

In summary, our results demonstrate the accessory protein ORF6 of SARS-CoV-2 imprisons mRNA in the nucleus, prevents nuclear import of a broad range of host factors, and strongly inhibits expression of newly transcribed transcripts via its interactions with the mRNA nuclear export factor Rae1 and the nuclear pore complex component Nup98. We hypothesize that the blockage of bidirectional nucleocytoplasmic transport by the *Sarbecovirus* accessory protein ORF6 likely leaves infected cells incapable of responding to the invading virus, allowing for the delayed host response and asymptomatic transmission observed in the current SARS-CoV-2 pandemic.

## MATERIALS AND METHODS

### Viral infection and oligo(dT) *in situ* hybridization.

Calu3 and HBEC3-KT-ACE2 cells were plated in μ-Slide VI 0.4 ibiTreated slides at densities of 30,000 and 50,000 cells per lane, respectively, and grown to 90% confluence. SARS-CoV-2/USA-WA1/2020 (NR-52281) was obtained from BEI Resources and propagated in Vero cells (USAMRIID). Calu3 and HBEC3-KT-ACE2 cells were mock infected or infected with SARS-CoV-2 at a multiplicity of infection (MOI) of 1 in Opti-MEM supplemented with 2% fetal bovine serum (FBS) for 1 h, and infection inoculum was replaced with Opti-MEM containing 2% FBS (Calu3) or airway epithelial growth medium (HBEC3-ACE2; PromoCell). Infection was performed within the biosafety level 3 (BSL3) facility at University of Washington following biosafety protocols. At 24 h postinfection (h.p.i.), cells were fixed with 4% paraformaldehyde in phosphate-buffered saline (PBS) at room temperature for 15 min and washed with PBS supplemented with SUPERaseIn RNase inhibitor.

The fixed cells were permeabilized with methanol and rehydrated in 70% ethanol followed by 1 M Tris-HCl (pH 8.0) (Invitrogen). The monolayer was then covered with hybridization buffer (1 mg/ml yeast tRNA, 0.005% bovine serum, 10% dextran sulfate and 25% formamide in 2× SSC buffer [1× SSC is 0.15 M NaCl plus 0.015 M sodium citrate]) containing an oligo(dT)(30) probe with an Alexa Fluor 594 fluorophore (IDT) attached to the 5′ end of the probe and incubated overnight at 37°C. The hybridization buffer was removed, and the cells were washed once with warmed 4× SSC buffer (Thermo Fisher), once with warmed 2× SSC buffer, and twice with room temperature 2× SSC buffer.

The cells were then blocked with 1% bovine serum in PBS containing 0.1% Tween 20 (PBST) for 30 min. To detect SARS-CoV-2-infected cells, the cells were incubated with an anti-SARS nucleocapsid protein antibody (1:200; clone 6H3; Abcam) for 1 h followed by an FITC-conjugated anti-mouse secondary antibody (1:1,000; Abcam). The cells were then mounted in Vectashield Vibrance antifade mounting medium with 4′,6′-diamidino-2-phenylindole (DAPI) (Vector Laboratories) and visualized with a Leica SP8X confocal microscope.

### Constructs and cloning.

The wild-type, N- and C-terminal mutant SARS-CoV-2 ORF6 constructs were amplified from double-stranded cDNA from a previously sequenced clinical SARS-CoV-2 isolate (WA12-UW8; EPI_ISL_413563) using the primers listed in Table S2 in the supplemental material. CloneAmp Hi-Fi PCR Premix (TaKaRa) and the following PCR conditions were used to generate the amplicons: 98°C for 2 min, followed by 35 cycles, with 1 cycle consisting of 98°C for 10 s, 55°C for 15 s, and 72°C for 30 s, followed by a final extension for 72°C for 5 min. ORF6 Δ22-30 was amplified from WA-UW-4572 (MT798143), and the matrix protein from vesicular stomatitis virus was amplified from pVSV eGFP dG (a gift from Connie Cepko; Addgene plasmid 31842) as described above using the primers listed in Table S2. A gBlock gene fragment (IDT) for ORF6 of SARS-CoV was synthesized based on the genome sequence of SARS-CoV isolate TW1 (GenBank accession no. AY291451.1). The resulting amplicons and gene fragment were then cloned into a modified pLenti CMV Puro plasmid (a gift from Eric Campeau & Paul Kaufman; Addgene plasmid 17448), which contains a 3′ WPRE sequence following the insert and a 3′ simian virus 40 (SV40) polyadenylation signal after the puromycin resistance cassette, with an N-terminal GFP or mCherry tag using the In-Fusion HD cloning kit (TaKaRa).

10.1128/mBio.00065-21.8 TABLE S2 Primers used in this study. Download Table S2, DOCX file, 0.01 MB. Copyright © 2021 Addetia et al. 2021 Addetia et al. https://creativecommons.org/licenses/by/4.0/ This content is distributed under the terms of the Creative Commons Attribution 4.0 International license.

For cloning of Rae1, STAT1, NR3C1, KPNA2, and KPNA3, RNA was extracted from 239T cells using the RNeasy Miniprep kit (Qiagen), and cDNA was synthesized using Superscript IV and oligo(dT) (IDT). The genes were then amplified from the resulting cDNA using the primers listed in Table S2 and CloneAmp Hi-Fi PCR Premix under the following PCR conditions: 98°C for 2 min, followed by 35 cycles, with 1 cycle consisting of 98°C for 10 s, 55°C for 15 s, and 72°C for 1 min, followed by a final extension for 72°C for 5 min. The resulting amplicon for Rae1 was cloned into a modified pcDNA4-TO vector with a C-terminal FLAG tag, the STAT1 amplicon was cloned into a modified pLenti CMV Puro plasmid with a C-terminal mCherry tag, and the remaining constructs were cloned into a modified pLenti CMV Puro plasmid with a N-terminal mCherry tag using the In-Fusion HD cloning kit.

### Specimen collection and whole-genome sequencing of SARS-CoV-2-positive clinical specimens.

Whole-genome sequencing of SARS-CoV-2-positive clinical specimens was conducted as part of an ongoing University of Washington Institutional Review Board-approved study (STUDY00000408) (38–41). Nasopharyngeal swabs were collected from patients suspected to have an infection with SARS-CoV-2 and stored in 3 ml of viral transport medium. RNA was extracted from 140 μl of medium using the Qiagen Biorobot. Sequencing libraries were prepared as previously described (34, 42). Briefly, RNA was treated with Turbo DNase (Thermo Fisher), and first-strand cDNA was synthesized using Superscript IV (Thermo Fisher) and random hexamers (IDT). Double-stranded cDNA was created using Sequenase version 2.0 (Thermo Fisher) and purified using 1.6× volumes of AMPure XP beads (Beckman-Coulter). Multiplex amplicon sequencing libraries were constructed using Swift Biosciences’ SARS-CoV-2 Multiplex Primer Pool and Normalase Amplicon kit and sequenced on a 2 × 300-bp run on an Illumina MiSeq.

A total of 712,394 sequencing reads were obtained for the clinical SARS-CoV-2 sample, WA-UW-4752. Sequencing reads were quality and adapter trimmed using Trimmomatic v0.38 (ILLUMINACLIP:TruSeq3-PE-SNAP.fa:2:30:10:1:true LEADING:3 TRAILING:3 SLIDINGWINDOW:4:30 MINLEN:75) (43) and aligned to the SARS-CoV-2 reference genome (NCBI reference sequence NC_045512.2) using BBMap version 38.70 (sourceforge.net/projects/bbmap/). Sequence reads were then clipped of synthetic PCR primers using Primerclip (https://github.com/swiftbiosciences/primerclip), and the final sequence alignment was visualized in Geneious version 11.1.4 (44).

The deletion identified within ORF6 of WA-UW-4752 was confirmed by reverse transcription-PCR and Sanger sequencing. For reverse transcription, single-stranded cDNA was constructed using Superscript IV. The resulting cDNA was used as the template for PCR with Phusion high-fidelity polymerase (Thermo Fisher) and the following primers: 5′-ATCACGAACGCTTTCTTATTAC-3′ and 5′-CTCGTATGTTCCAGAAGAGC-3′. PCR was conducted using the following conditions: 98°C for 30 s, followed by 35 cycles, with 1 cycle consisting of 98°C for 10 s, 55°C for 15 s, and 72°C for 30 s, followed by a final extension at 72°C for 5 min. The resulting amplicons were run on a 2% agarose gel, extracted from the gel using the QIAquick gel extraction kit (Qiagen), and Sanger sequenced by Genewiz, Inc., with the same primers used for PCR.

Other strains with the same deletion in ORF6 were identified by querying GISAID (accessed 17 July 2020). The genetic relatedness of these strains was assessed by aligning the genomes of these strains as well as 110 other global clinical SAR-CoV-2 strains using MAFFT v7.453 (45). A phylogenetic tree was generated using RAxML version 8.2.11 (46) and visualized with R (version 3.6.1) using the ggtree package (47). Strains were further classified using the web-based lineage assigner, Pangolin (https://pangolin.cog-uk.io/) (48).

### Cell culture and oligo(dT) *in situ* hybridization of transiently transfected cells.

293T cells were maintained in Dulbecco’s modified Eagle’s medium (DMEM) (GE Healthcare Life Sciences) supplemented with 10% FBS (Sigma-Aldrich), 1× HEPES (Thermo Fisher), and 1× GlutaMAX (Thermo Fisher) (293T medium). A549 and Calu3 cells were maintained in Dulbecco’s modified Eagle’s medium (GE Healthcare Life Sciences) supplemented with 10% FBS (Sigma-Aldrich) with 1× penicillin-streptomycin (Thermo Fisher). HBEC3-KT cells (ATCC CRL-4051) stably expressing human ACE2 were maintained in airway epithelial cell growth medium (PromoCell) supplemented with 1× penicillin-streptomycin. HBEC3-KT cells were infected with pseudotyped lentivirus packaging human ACE2 in the presence of 8 μg/ml Polybrene. Lentivirus was generated by cotransfecting 293T cells with the pHAGE2-ACE2-WT plasmid (BEI Resources, catalog no. NR-52512), and lentiviral helper plasmids pMD2.G (Addgene plasmid 12259), pHDM-Hgpm2 (BEI Resources, catalog no. NR-52517), pHDM-tat1b (BEI Resources, catalog no. NR-52518), and pRC-CMV-Rev1b (BEI Resources, catalog no. NR-52519). Cells with high expression levels of ACE2 were collected via fluorescence-activated cell sorting via cell staining with labeled anti-human ACE2 antibody (Abcam) and pooled for further expansion (HBEC3-ACE2).

293T, A549, or Calu3 cells were plated in μ-Slide eight-well ibiTreated chamber slides at a density of 50,000 to 120,000 cells per well and grown overnight to 50 to 90% confluence. 293T cells were transfected with 300 ng of plasmid DNA using a 3:1 ratio of PEI MAX (Polysciences) in Opti-MEM (Thermo Fisher). A549 and Calu3 cells were transfected with 250 ng of plasmid DNA using Lipofectamine 3000 (Thermo Fisher) diluted in Opti-MEM. All three cell lines were incubated for 24 h posttransfection. The cells were then washed with PBS (pH 7.4; without Ca^2+^ or Mg^2+^) (Thermo Fisher) and fixed with 4% paraformaldehyde. Oligo(dT) *in situ* hybridization was performed as described above. The cells were then blocked with 1% bovine serum in PBS containing 0.1% Tween 20 (PBST) for 1 h. To detect the GFP-tagged proteins, the cells were incubated with a FITC-conjugated anti-GFP antibody (1:1,000; Abcam) for 1 h. The antibody was removed, and the cells were washed three times with PBST. The cells were mounted in Vectashield Vibrance antifade mounting medium with DAPI and visualized with a Leica SP8X confocal microscope.

### Measurement of nascent protein synthesis.

293T cells were plated at a density of 15,000 cells per well in poly-l-lysine-coated 96-well, clear-bottom, opaque-walled plates and grown overnight until they reached approximately 70% confluence. The cells were then transfected with 70 ng of mCherry-tagged constructs using a 3:1 ratio of PEI MAX in Opti-MEM and incubated for 24 h. The cells were then washed twice with the DMEM containing no methionine (Thermo Fisher) and supplemented with 1× GlutaMAX and 200 nM l-cystine (Sigma) and incubated in this medium for 30 min.

Nascent protein synthesis was measured using the Click-iT AHA Alexa Fluor 488 Protein Synthesis HCS assay kit (Thermo Fisher) following the manufacturer’s recommendations. In brief, cells were incubated in DMEM containing no methionine and supplemented with 1× GlutaMAX, 200 nM l-cystine, and 50 μM Click-iT AHA reagent for 2 h. A control condition in which 2 μM puromycin was added to the labeling solution was included for each replicate. The cells were fixed with 4% paraformaldehyde and permeabilized with 0.5% Triton X-100 (Sigma). Nascent proteins which incorporated the Click-iT AHA reagent were then FITC tagged using the Click-iT reaction cocktail. The nuclei were then stained with Hoechst 33342, and the FITC and Hoechst 33342 fluorescent values in each well were measured with a Victor Nivo plate reader (Perkin Elmer). The relative level of nascent protein synthesized between each condition was determined by calculating the FITC/Hoechst 33342 ratio. Differences in the mean FITC/Hoechst 33342 ratio between experimental conditions were assessed in R using the unpaired *t* test.

### ORF6-mCherry transient cotransfections.

Transient cotransfections with GFP-tagged constructs and a modified pLenti CMV Puro vector encoding the fluorescent reporter mCherry were conducted in six-well plates. The day prior to transfection, 500,000 293T cells were plated into each well of the six-well plate and grown overnight until they reached approximately 50% confluence. The cells were then transfected with 2 μg of GFP-tagged construct and 2 μg of mCherry using a 3:1 ratio of PEI MAX in Opti-MEM. A549 cells were plated into six-well plates at a density of 500,000 cells per well and grown overnight until they reach approximately 85% confluence. The A549 cells were then transfected with 1.5 μg of GFP-tagged construct and 1.5 μg of mCherry using Lipofectamine 3000 diluted in Opti-MEM. Cells were incubated for 24 to 48 h following transfection and visualized using the EVOS M5000 imaging system (Thermo Fisher) with GFP and Texas Red filter cubes.

mCherry fluorescence intensities were measured with ImageJ v1.53a by an individual blinded to experimental design. All images were 8-bit grayscale and 2,048 × 1,536 (3.1 megapixels). Background thresholds were set at the same level across all images, and mean fluorescence intensities of regions of interest greater than 200 pixels were calculated. Three fields were analyzed for each experimental condition. The mean fluorescent intensity for each field was calculated after adjusting for background fluorescence signal and normalized to the control condition. Differences in mean fluorescent intensities between experimental conditions were assessed in R using the unpaired *t* test.

Cell lysates were collected 24 to 48 h after transfection using radioimmunoprecipitation assay (RIPA) buffer (Thermo Fisher). The total protein content was measured using the Pierce BCA protein assay kit (Thermo Fisher), and 7.5 μg of lysate was run on a 4 to 12% Bis-Tris sodium dodecyl sulfate (SDS)-polyacrylamide gel with morpholinepropanesulfonic acid (MOPS) running buffer (Invitrogen) under reducing conditions. The samples were then transferred to a 0.45-μm nitrocellulose membrane using the XCell Blot II module (Invitrogen). Blotting was performed using the following primary antibodies: 1:1,000 anti-GFP (Cell Signaling; clone 4B10), 1:500 anti-mCherry (Cell Signaling; clone E5D8F), and 1:1,000 anti-α-tubulin (Cell Signaling; clone DM1A), which was followed by staining with either 1:10,000 IRDye 680RD anti-mouse IgG secondary antibody (Licor) or 1:5,000 IRDye 800CW anti-Rabbit IgG secondary antibody (Licor). Blots were then visualized on a Licor Odyssey imager using Image Studio version 2.0.

### Affinity purification of GFP-tagged constructs.

The day prior to transient transfection, 10-cm plates were seeded with 4 × 10^6^ 293T cells and grown overnight to approximately 50% confluence. The cells were transfected with 7 μg of plasmid DNA using a 3:1 ratio of PEI MAX in Opti-MEM. Forty-four to 48 h after transfection, the cells were washed with PBS and collected using PBS containing 0.1 mM EDTA. The cells were pelleted, resuspended in 500 μl TEN (50 mM Tris [pH 8.0], 150 mM NaCl, and 1 mM EDTA) buffer with 0.5% NP-40 and lysed by rotation for 45 to 60 min at 4°C. The lysates were centrifuged at 13,000 RPM for 5 min at 4°C, and the supernatant was transferred to a new tube and cleared of residual IgG by rotation with Protein G Sepharose 4 Fast Flow (GE Healthcare Life Sciences) for 30 min at 4°C. Cleared lysates were transferred to new tubes and incubated overnight at 4°C with anti-GFP Nanobody Affinity gel (BioLegend). The affinity gel was then pelleted and washed twice using TEN buffer with 0.1% NP-40 and resuspended in equal volumes of NuPage LDS sample buffer (Thermo) containing 143 mM 2-mercaptoethanol (Sigma-Aldrich). Western blotting using the elutes from affinity purification and the prepurified input lysates were performed as described above with the following primary antibodies: 1:1,000 anti-GFP, 1:1,000 anti-α-tubulin, 1:2,000 anti-Rae1 (Abcam; clone EPR6923), and 1:1,000 anti-Nup98 (Abcam; clone 2H10).

### Rae1 rescue of mCherry expression.

293T cells were plated in six-well plates at a seeding density of 500,000 cells per well and grown overnight until they reached approximately 50% confluence. Cells were then transfected with 0.5 μg of the GFP-SARS-CoV-2 wild-type ORF6 construct, 0.5 μg of mCherry, and 0, 0.25, 0.5, 1, or 2 μg of Rae1-FLAG using a 3:1 ratio of PEI MAX in Opti-MEM. GFP expression and mCherry expression were visualized 44 to 48 h following transfection using the EVOS M5000 imaging system with GFP and Texas Red filter cubes. Western blotting was performed as described above with the following primary antibodies: 1:1,000 anti-GFP, 1:500 anti-mCherry, 1:1,000 anti-α-tubulin, and 1:1,000 anti-FLAG (Sigma; clone M2).

### Nuclear import assays.

For all nuclear import assays, 293T cells were plated at a density of 50,000 cells per well in μ-Slide eight-well ibiTreated chamber slides and incubated overnight. STAT1 nuclear import was analyzed by cotransfecting cells with 150 ng of GFP-tagged constructs and 150 ng of a STAT1 construct containing a C-terminal mCherry tag. The transfected cells were incubated for 24 h and stimulated with 100 IU/ml recombinant human interferon beta (R&D Systems) for 1 h. Glucocorticoid receptor nuclear import was analyzed by cotransfecting cells with 150 ng of GFP-tagged constructs and 150 ng of an mCherry-tagged glucocorticoid receptor construct. The cells were incubated for 24 h and stimulated with 100 nM dexamethasone (Sigma) for 30 min. KPNA2 and KPNA3 localization patterns were analyzed by cotransfecting cells with 150 ng of GFP-tagged constructs and 150 ng of mCherry-tagged KPNA2 or KPNA3 constructs. The localization patterns were visualized 24 h posttransfection. All wells were fixed with 4% paraformaldehyde, mounted in in Vectashield Vibrance antifade mounting medium with DAPI, and visualized with a Leica SP8X confocal microscope.

### Data availability.

Sequencing reads and genome assemblies are available under NCBI BioProject accession no. PRJNA610428.
