# Supplementary material for: SARS-CoV-2 ORF6 Disrupts Bidirectional Nucleocytoplasmic Transport through Interactions with Rae1 and Nup98
Source: mBio. 2021 Apr 13;12(2):e00065-21. doi: 10.1128/mBio.00065-21 (PMC8092196; doi:10.1128/mBio.00065-21)
Supplement: FIG S1 [file mBio.00065-21-sf001.pdf]

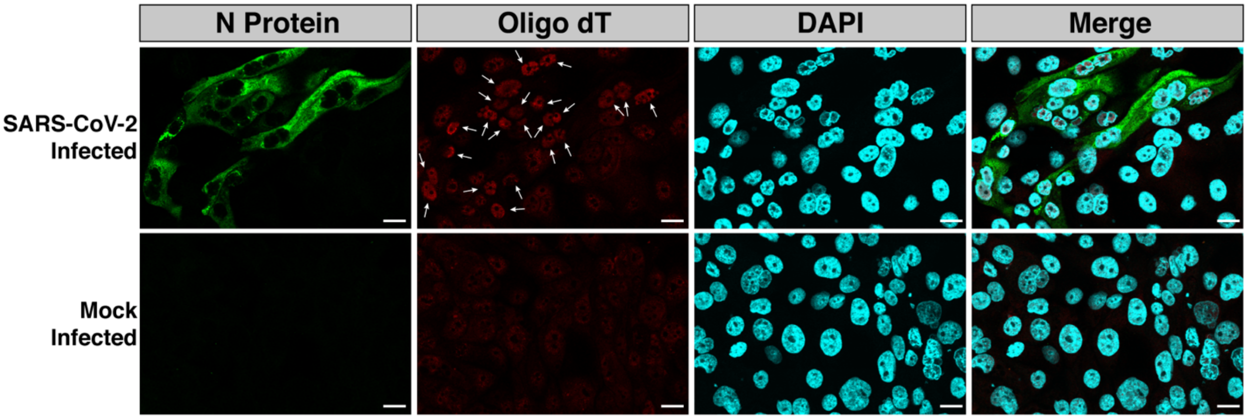

Figure S1. Poly-A mRNA staining of Calu3 cells infected with SARS-CoV-2 revealed nuclear localization of mRNA in infected cells (highlighted with white arrows). Scale bar: 20 μm.
