# Supplementary material for: SARS-CoV-2 ORF6 Disrupts Bidirectional Nucleocytoplasmic Transport through Interactions with Rae1 and Nup98
Source: mBio. 2021 Apr 13;12(2):e00065-21. doi: 10.1128/mBio.00065-21 (PMC8092196; doi:10.1128/mBio.00065-21)
Supplement: FIG S2 [file mBio.00065-21-sf002.pdf]

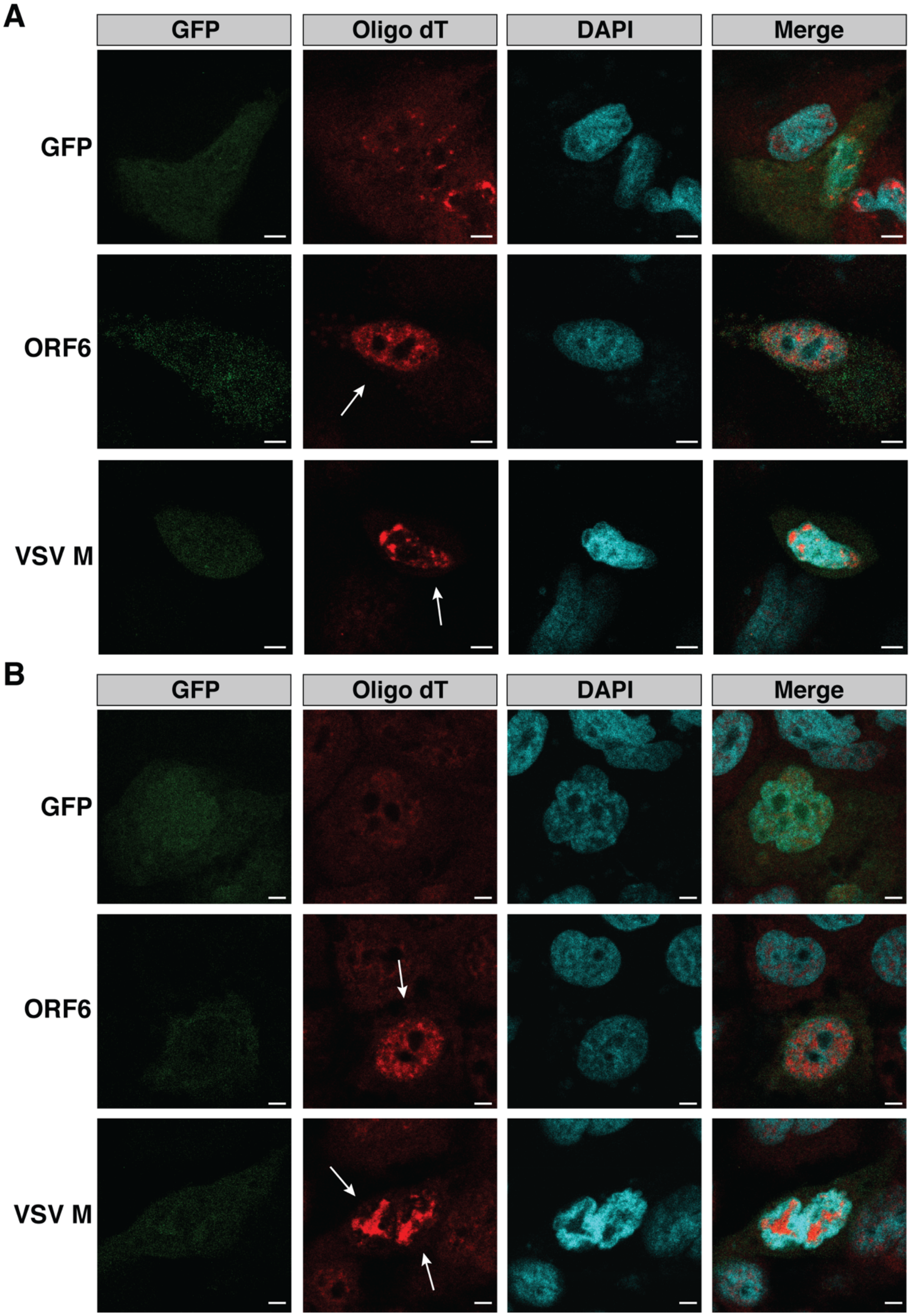

Figure S2. A) A549 and B) Calu3 cells were transiently transfected with GFP, GFP-SARS-CoV-2 ORF6, or GFP-VSV M. Staining for poly-A mRNA revealed cells expressing SARS-CoV-2 ORF6 and VSV M accumulated mRNA in the nuclei. White arrows depict cells transfected with SARS-CoV-2 ORF6 or VSV M. Scale bar: 5 μm.
