# Supplementary material for: SARS-CoV-2 ORF6 Disrupts Bidirectional Nucleocytoplasmic Transport through Interactions with Rae1 and Nup98
Source: mBio. 2021 Apr 13;12(2):e00065-21. doi: 10.1128/mBio.00065-21 (PMC8092196; doi:10.1128/mBio.00065-21)
Supplement: FIG S3 [file mBio.00065-21-sf003.pdf]

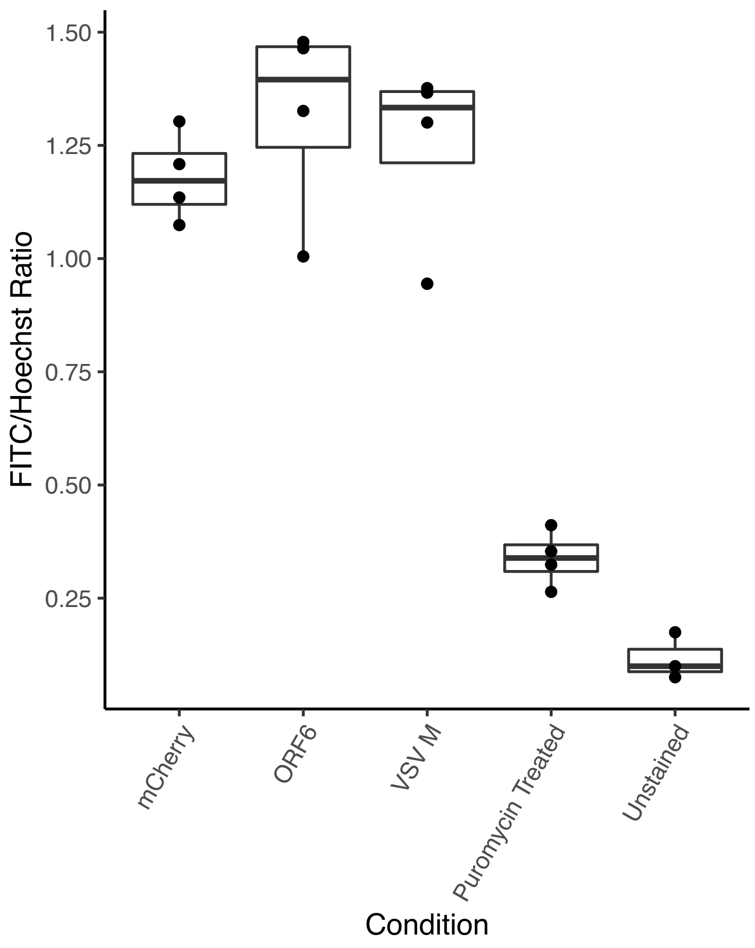

Figure S3. Nascent protein synthesis was measured in cells transfected with mCherry, SARS-CoV-2 ORF6, or VSV M and puromycin treated cells using the Click-iT AHA Alexa Fluor 488 Protein Synthesis HCS Assay. Similar levels of nascent protein synthesis were observed in mCherry, SARS-CoV-2 ORF6, and VSV M-expressing cells.
