# Supplementary material for: SARS-CoV-2 ORF6 Disrupts Bidirectional Nucleocytoplasmic Transport through Interactions with Rae1 and Nup98
Source: mBio. 2021 Apr 13;12(2):e00065-21. doi: 10.1128/mBio.00065-21 (PMC8092196; doi:10.1128/mBio.00065-21)
Supplement: FIG S4 [file mBio.00065-21-sf004.pdf]

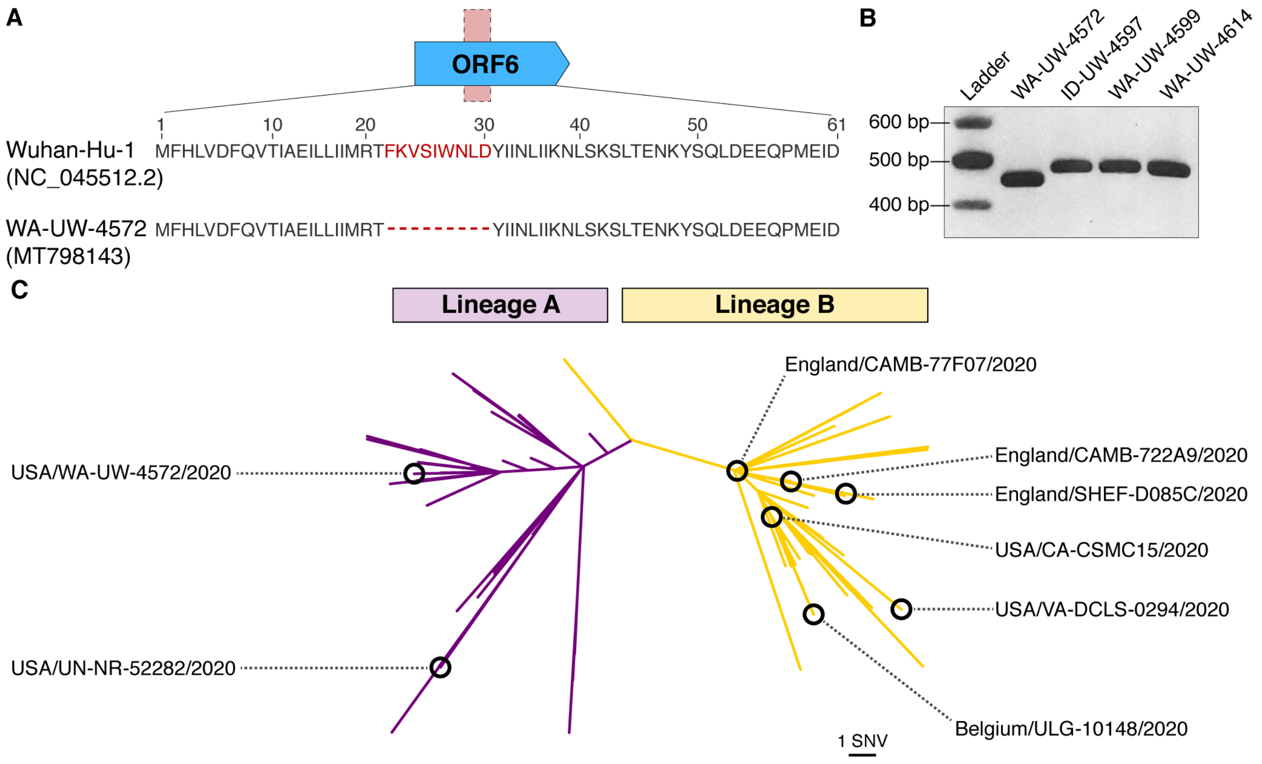

Figure S4. Multiple clinical SARS-CoV-2 isolates and a serially passaged cultured SARS-CoV-2 isolate contain a 9-amino acid deletion in ORF6. A) Schematic representation of the 9-amino acid deletion in ORF6 identified through whole genome sequencing of the SARS-CoV-2 clinical isolate, WA-UW-4572. B) RT-PCR with primers spanning ORF6 yielded a 452 bp PCR product for WA-UW-4572 rather than a 479 bp product confirming the ORF6 deletion in WA-UW-4572. C) Six other clinical isolates and a cultured isolate with an identical deletion in ORF6 were identified by analyzing the ORF6 sequences of 67,000 publicly available SARS-CoV-2 genomes. The isolates were genetically distinct and belonged to both major SARS-CoV-2 lineages.
