# Supplementary material for: SARS-CoV-2 ORF6 Disrupts Bidirectional Nucleocytoplasmic Transport through Interactions with Rae1 and Nup98
Source: mBio. 2021 Apr 13;12(2):e00065-21. doi: 10.1128/mBio.00065-21 (PMC8092196; doi:10.1128/mBio.00065-21)
Supplement: TABLE S1 [file mBio.00065-21-st001.docx]

| **Strain Name** | **Lineage** | **Collection Location** | **Accession Number** |
| --- | --- | --- | --- |
| Belgium/ULG-10148/2020 | B.1 | Belgium | EPI_ISL_447145 |
| England/CAMB-722A9/2020 | B.1 | England, UK | EPI_ISL_439593 |
| England/CAMB-77F07/2020 | B.1 | England, UK | EPI_ISL_441819 |
| England/SHEF-D085C/2020 | B.1.5 | England, UK | EPI_ISL_475487 |
| USA/CA-CSMC15/2020 | B.1 | California, USA | EPI_ISL_475624 |
| USA/UN-NR-52282/2020 | A | Cell culture isolate | EPI_ISL_456656 |
| USA/VA-DCLS-0294/2020 | B.1 | Virginia, USA | EPI_ISL_463097 |
| USA/WA-UW-4572/2020 | A.1 | Washington, USA | MT798143 |

**Table S1.** Clinical and cultured SARS-CoV-2 isolates with a 9 amino acid deletion in ORF6 identified by analyzing ORF6 sequences from over 67,000 SARS-CoV-2 strains (<https://www.gisaid.org/>; accessed July 17, 2020).
