# Supplementary material for: SARS-CoV-2 ORF6 Disrupts Bidirectional Nucleocytoplasmic Transport through Interactions with Rae1 and Nup98
Source: mBio. 2021 Apr 13;12(2):e00065-21. doi: 10.1128/mBio.00065-21 (PMC8092196; doi:10.1128/mBio.00065-21)
Supplement: FIG S5 [file mBio.00065-21-sf005.pdf]

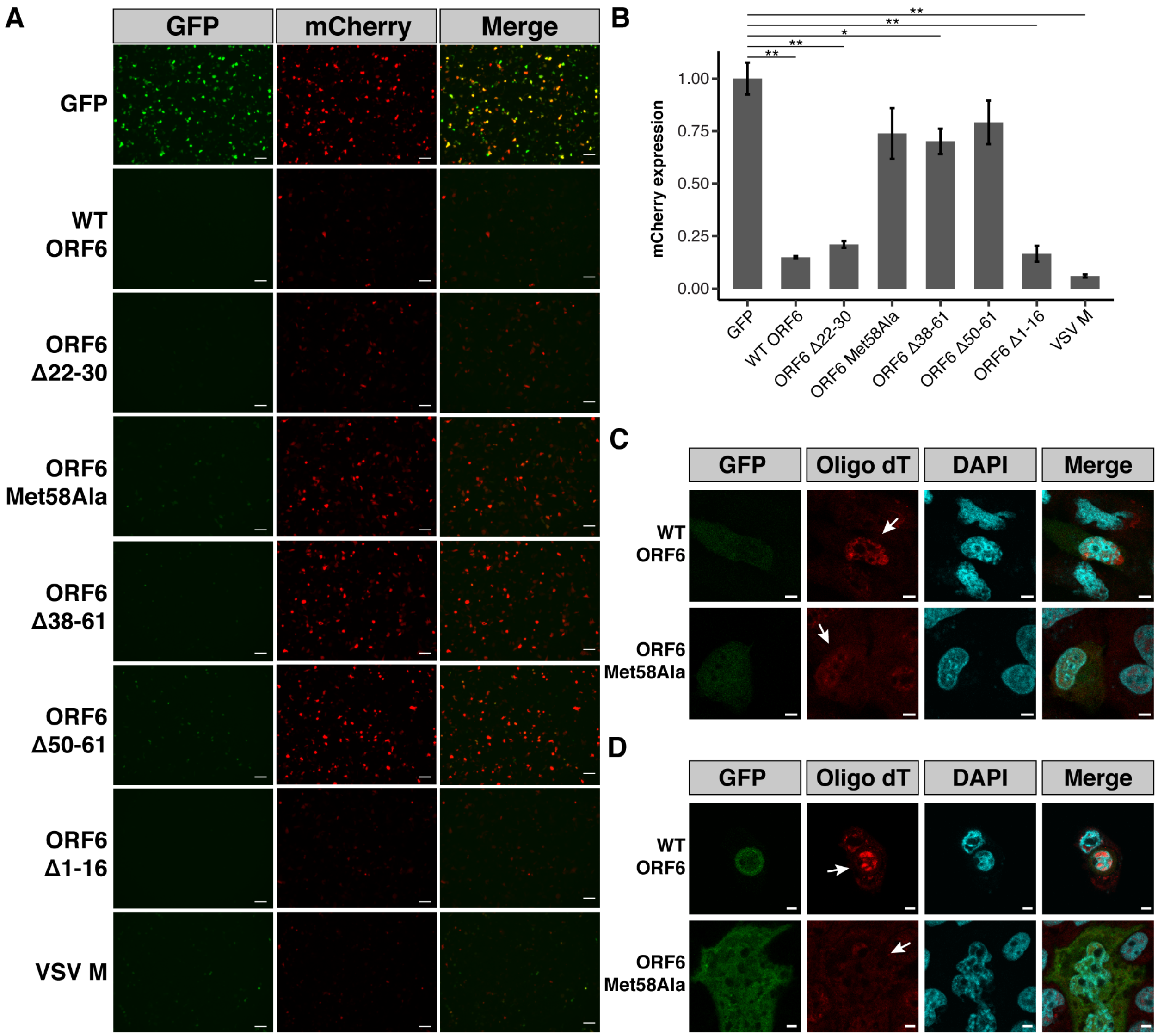

Figure S5. A) A549 cells were transiently transfected with GFP-tagged constructs and mCherry. Cells were visualized 24 hours after transfection and all images were taken with identical fluorescence gain settings. Scale bar: 100 µm. B) Fluorescent intensities across 3 fields per conditions were measured with ImageJ and displayed as mean ± standard error. Staining for poly-A mRNA in C) A549 and D) Calu3 cells revealed SARS-CoV-2 ORF6 Met58Ala-expressing cells do not accumulate mRNA in the nuclei, consistent with the results of the mCherry co-transfection assay. White arrows indicate transfected cells. Scale bar: 5 µm. * $p < 0.05$; ** $p < 0.01$.
