# Supplementary material for: SARS-CoV-2 ORF6 Disrupts Bidirectional Nucleocytoplasmic Transport through Interactions with Rae1 and Nup98
Source: mBio. 2021 Apr 13;12(2):e00065-21. doi: 10.1128/mBio.00065-21 (PMC8092196; doi:10.1128/mBio.00065-21)
Supplement: FIG S6 [file mBio.00065-21-sf006.pdf]

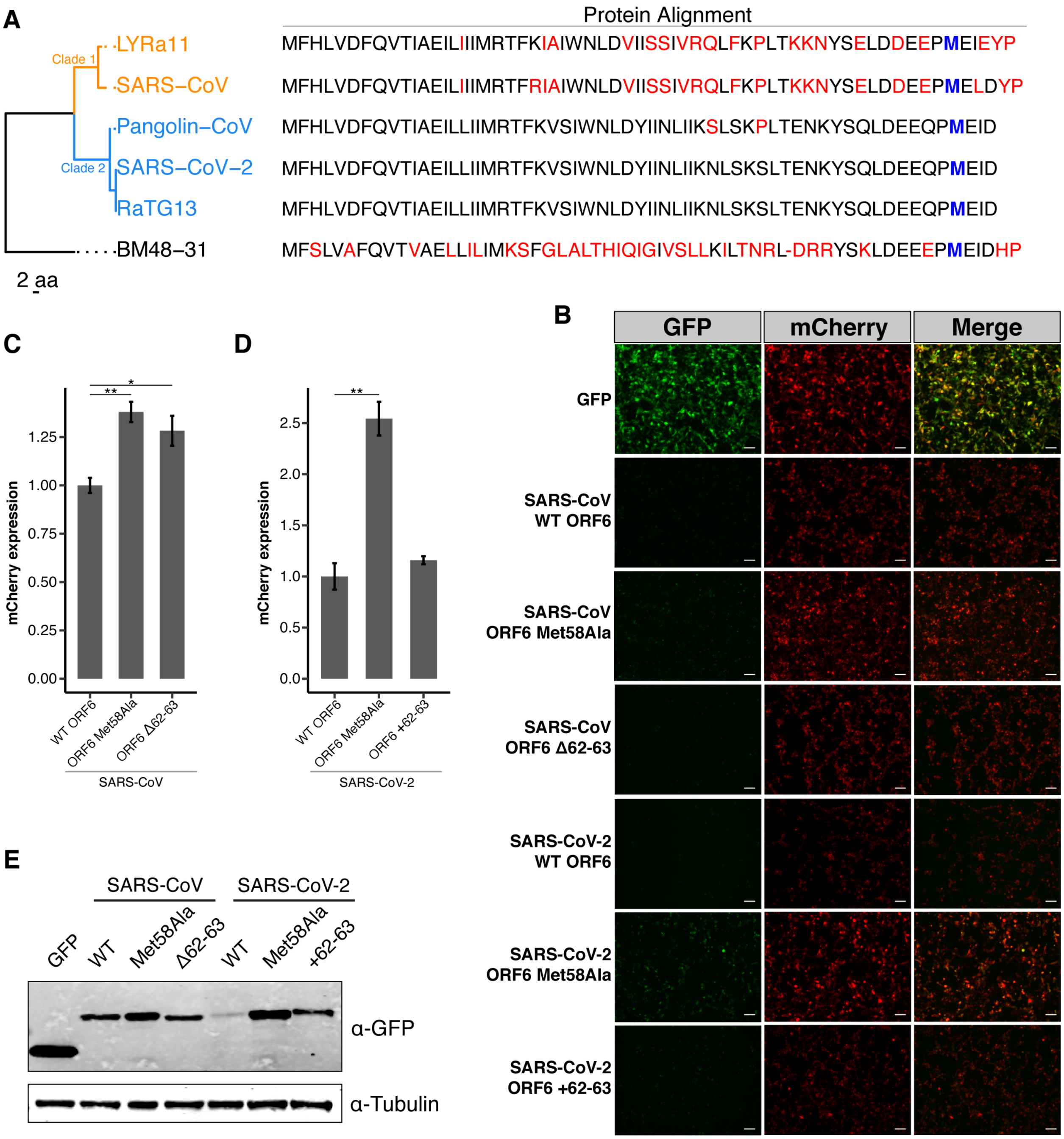

Figure S6. A) An analysis of the ORF6 homologs in the related zoonotic sarbecoviruses, LYRa11 (KF569996.1), Pangolin-CoV (EPI_ISL_410721), and RaTG13 (MN996532.2), reveals the ORF6 protein in the Clade 1 sarbecoviruses is 2 amino acid longer than that in the Clade 2 sarbecoviruses. The Clade 3 sarbecovirus BM48-31 (NC_014470.1) was used as an outgroup in the phylogenetic analysis. Residues different from SARS-CoV-2 ORF6 are colored red while the conserved methionine residue is colored blue. B) 293T cells were transiently transfected with GFP-tagged constructs and mCherry. Expression was visualized 48 hours after transfection and fluorescent intensities across 3 fields per experimental condition were measured for C)

SARS-CoV ORF6 constructs and D) SARS-CoV-2 ORF6 constructs. Scale bar: 100 µm. * $p < 0.05$; ** $p < 0.01$. E) Expression levels of the GFP-tagged SARS-CoV and SAR-CoV-2 ORF6 constructs were visualized by western blotting.
