# Supplementary material for: SARS-CoV-2 ORF6 Disrupts Bidirectional Nucleocytoplasmic Transport through Interactions with Rae1 and Nup98
Source: mBio. 2021 Apr 13;12(2):e00065-21. doi: 10.1128/mBio.00065-21 (PMC8092196; doi:10.1128/mBio.00065-21)
Supplement: TABLE S2 [file mBio.00065-21-st002.docx]

**Table S2.** Primers used in this study.

| **Name** | **Sequence (5´ to 3´)** |
| --- | --- |
| SARS-CoV-2-ORF6-pLenti-F | GCAGCGGTGGCGGTGGATCCATGTTTCATCTCGTTGACTTTC |
| SARS-CoV-2-ORF6-pLenti-R | TCCAGAGGTTGATTGTCGACGCGCCCGGGTTAATCAATCTCCATTGGTTGC |
| SARS-CoV-2-ORF6-M58A-pLenti-R | TCCAGAGGTTGATTGTCGACGCGCCCGGGTTAATCAATCTCCGCTGGTTGCTCTTCAT |
| SARS-CoV-2-ORF6-del38-61-pLenti-R | TCCAGAGGTTGATTGTCGACGCGCCCGGGTTAAATTATGAGGTTTATGATGTAATC |
| SARS-CoV-2-ORF6-del50-61-pLenti-R | TCCAGAGGTTGATTGTCGACGCGCCCGGGTTAATATTTATTCTCAGTTAGTGAC |
| SARS-CoV-2-ORF6-del1-16-pLenti-R | GCAGCGGTGGCGGTGGATCCATTATTATGAGGACTTTTAAAG |
| SARS-CoV-2-ORF6-add62-63-pLenti-R | ATTGTCGACGCGCCCGGGTTATGGATAATCAATCTCCATTGGTTGCTC |
| SARS-CoV-ORF6-pLenti-F | CGGTGGCGGTGGATCCATGTTTCATCTTGTTGACTTCC |
| SARS-CoV-ORF6-M58A-pLenti-R | ATTGTCGACGCGCCCGGGTTATGGATAATCTAACTCCGCAGGTTC |
| SARS-CoV-ORF6-del62-63-pLenti-R | ATTGTCGACGCGCCCGGGTTAATCTAACTCCATAGGTTCTTC |
| VSV-M-pLenti-F | GCAGCGGTGGCGGTGGATCCATGAGTTCCTTAAAGAAGATTCTC |
| VSV-M-pLenti-R | TCCAGAGGTTGATTGTCGACGCGCCCGGGTCATTTGAAGTGGCTGATAGAATCC |
| Rae1-pCDNA4TO-FLAG-F | TACCGAGCTCGGATCCATGAGCCTGTTTGGAACAACC |
| Rae1-pCDNA4TO-FLAG-R | CACCGCCTCCCTCGAGCTTCTTATTCCTGGGCTTTAGC |
| STAT1-pLenti-F | GACACCGACTCTAGAGCCACCATGTCTCAGTGGTACGAACTTC |
| STAT1-pLenti-R | GCGACCGGTGGATCCGGTACTGTGTTCATCATACTGTCGAA |
| GR-pLenti-F | CGGTGGCGGTGGATCCATGGACTCCAAAGAATCATTAACTC |
| GR-pLenti-FR | ATTGTCGACGCGCCCGGGTCACTTTTGATGAAACAGAAG |
| KPNA2-pLenti-F | CGGTGGCGGTGGATCCATGTCCACCAACGAGAATG |
| KPNA2-pLenti-R | CTCCTGGGACCTTTAACTTTTAGCCCGGGCGCGTCGACAAT |
| KPNA3-pLenti-F | CGGTGGCGGTGGATCCATGGCCGAGAACCCCAG |
| KPNA3-pLenti-R | CAAACAAAAGAATTTAATTTTTAACCCGGGCGCGTCGACAAT |
